# *De novo* assembly of wheat root transcriptomes and transcriptional signature of longitudinal differentiation

**DOI:** 10.1371/journal.pone.0205582

**Published:** 2018-11-05

**Authors:** Ghana Shyam Challa, Wanlong Li

**Affiliations:** 1 Department of Biology and Microbiology, South Dakota State University, Brookings, SD, United States of America; 2 Department of Plant Science, South Dakota State University, Brookings, SD, United States of America; Youngstown State University, UNITED STATES

## Abstract

Hidden underground, root systems constitute an important part of the plant for its development, nourishment and sensing the soil environment around it, but we know very little about its genetic regulation in crop plants like wheat. In the present study, we *de novo* assembled the root transcriptomes in reference cultivar Chinese Spring from RNA-seq reads generated by the 454-GS-FLX and HiSeq platforms. The FLX reads were assembled into 24,986 transcripts with completeness of 54.84%, and the HiSeq reads were assembled into 91,543 high-confidence protein-coding transcripts, 2,404 low-confidence protein-coding transcripts, and 13,181 non-coding transcripts with the completeness of >90%. Combining the FLX and HiSeq assemblies, we assembled a root transcriptome of 92,335 ORF-containing transcripts. Approximately 7% of the coding transcripts and ~2% non-coding transcripts are not present in the current wheat genome assembly. Functional annotation of both assemblies showed similar gene ontology patterns and that ~7% coding and >5% non-coding transcripts are root-specific. Transcription quantification identified 1,728 differentially expressed transcripts between root tips and maturation zone, and functional annotation of these transcripts captured a transcriptional signature of longitudinal development of wheat root. With the transcriptomic resources developed, this study provided the first view of wheat root transcriptome under different developmental zones and laid a foundation for molecular studies of wheat root development and growth using a reverse genetic approach.

## Introduction

As the “hidden half” of a plant, root systems provide plant water, nutrients, and an anchorage from the soil, produce growth regulators and sense soil environmental changes such as pH, moisture, and mineral content. A well-developed root system is critical for sustainable crop production. Despite the important roles in plant development and growth, our understanding of root development and growth is still very limited as compared to the aboveground half. Nevertheless, most knowledge of root biology comes from the model plant Arabidopsis. Rich genomic resources, non-soil cultivation and anatomical simplicity make the Arabidopsis root state of the art in plant biology at both molecular and cellular levels, including identification of many genes involving various aspects of root development, characterization of hormone interaction, cell type definition, and environmental responses [1]. Dicots and monocots differ significantly in root system architecture and cellular organization. Compared to the tap root system in dicots, monocot roots are fibrous with large quiescent centers, the separate origin of endodermis and cortex in ground tissue, multiple layers of cortical cells with variable cell numbers, and multiple-tissue occurrence of lateral roots [2]. With a finished genome and relative ease of genetic transformation, rice has emerged a model for grass root biology study and has proven to be informative [3, 4]. In contrast, very little information is available in the small-grain crops, including barley, oats, rye, and wheat, which grow in relatively dry conditions and have large genomes.

Common wheat or bread wheat (*Triticum aestivum* L., genomes AABBDD) is a hexaploid species of relatively recent origin and one of the most important food sources, providing ~20% daily caloric consumption. As the most widely adapted crop, wheat plays an important role in the global food security. Mainly due to climate change, however, wheat production is facing numerous challenges from biotic stress and abiotic stress. Understanding the molecular mechanisms underlying root development, growth and the environmental response is a prerequisite for improving tolerance to the soil-borne stress, such as drought and waterlogging, using biotech approaches. Functional genomics has long been expected to play an important role in wheat root studies. Of the ~1.3 million wheat expressed sequence tags (ESTs) from 147 complementary DNA (cDNA) libraries, 26,849 ESTs of 25 cDNA libraries were made from the root tissues of reference genotype Chinese Spring (CS; http://ncbi.nlm.nih.gov). But they are far apart from covering the root transcriptome, particularly for those transcripts that are low in abundance but important in function, such as transcription factors (TFs). Compared to the traditional EST development and microarray hybridization, RNA-Seq offers unprecedented capacity and resolution in revealing the landscape and dynamics of complex transcriptomes. As the sequencing cost continues to drop, RNA-Seq has been the favorite choice for transcriptome analysis of the non-model plant species [5]. Without finished genome sequences, the transcriptomes of the non-model species are assembled *de novo*. Although draft genome sequences of common wheat cultivar Chinese Spring (CS) [6, 7] and the A-genome [8] and D-genome progenitors of wheat [9] were reported recently, their utility in transcriptome analysis remains to be tested. Many RNA analyses have been performed in wheat [10–28]. Half of these researches assembled the transcriptomes *de novo* due to lack of a finished wgeat genome assembly. In another aspect, a *de novo* transcriptome assembly also benefits annotation of the wheat genome by identifying the novel genes that are not included in the current gene models and improving the current annotation.

Most of the transcriptomes assembled were mainly for aboveground tissues such as the embryogenic callus [27], developing grains [10, 20], spikes [16], microspores [15], leaves [18, 19, 23], or mixed tissues from whole plants [12]. No transcriptomes have been assembled for root development study. To gain a global view of the allelic interaction and its effect on the root transcriptome at large and to lay a foundation for root functional genomics, we initiated a wheat root transcriptome project. As the first stage, we sequenced the three root RNA samples of CS using 454 GS-FLX (Roche, Branford, CT, USA) and HiSeq (Illumina, San Diego, CA, USA) platforms. Assembly and quantification of the wheat root transcriptomes provide the first view of the transcriptional landscape of wheat root development. Here, we report the *de novo* assembly of the root transcriptomes, characterization of the assembled transcripts, expression profiling of the genes in the root tip and the mature part of the roots, and their implication to wheat root development and growth.

## Material and methods

### Plant material and RNA extraction

Root tissues collected from two separate germination experiments for RNASeq analysis by 454/Roche and Illumina sequencing platforms. In experiment 1, ~100 CS seeds were germinated on the tap water-wetted paper towels in a polystyrene container with lid (4 5/16” x 4 5/16” x 1 1/8”; Hoffman Manufacturing, Inc, Corvallis, OR), and 3-mm root tips were harvested from the 3 day old seedlings and frozen in liquid nitrogen immediately. In experiment 2, the CS seeds were sown in deep pots containing steriled sands, and root tips of ~3mm (mainly meristematic zone) and rest of the roots (mainly the maturation zone) were collected from seven day-old seedlings separately andfrozen in liquid nitrogen immediately. Three biological replicates were included for each developmental zone. Total RNA was extracted using Trizol (Thermo Fisher Scientific, Waltham, MA) following the manufacturer’s instruction. The RNA sample from the experiment 1 was purified using a mRNA-only kit (Epicentre, Madison, WI, USA) for message RNA (mRNA), and the RNA samples from the experiment 2 were purified using the RNeasy mini kit (Qiagen, Valencia, CA) following the manufacturer’s instruction. Concentration and integrity of the purified RNA samples were quantified using an Agilent 2100 Bioanalyzer (Agilent Technologies, Palo Alto, CA), and samples with an RNA integrity number (RIN) greater than eight were used in the subsequent analyses.

### 454 GS Titanium FLX sequencing

The purified mRNA from the experiment 1 was submitted to the Integrated Genomics Facility at Kansas State University, Manhattan, KS, for cDNA synthesis using random primers, for construction of a DNA sequencing library using a standard cDNA rapid library construction kit from 454/Roche and a sequencing run on a 454/Roche Titanium platform.

### Illumina sequencing

RNA samples extracted from root tips and rest of the root tissue (mainly maturation zone) from plants grown in experiment 2 were submitted the DNA Core Facility at University of Missouri, Columbia, MO, for cDNA synthesis, sequencing library construction and sequencing. Six barcoded sequencing libraries for three biological replicates for the meristematic zone and three biological replicates for the maturation zone were prepared using the TruSeq RNA Library Prep Kit (Illumina). These six libraries were pooled and sequenced in one lane on the HiSeq 2000 platform (Illumina) to generate 100 bp single-end reads.

### Quality control and preprocessing

Adapter sequences used during the library preparation were trimmed from the 454-GS-FLX reads using a Perl script from NGSQC toolkit [29]. The HiSeq reads were trimmed by a Java-based program Trimmomatic [30] using the -phred33 and the reads shorter than the minimum length cutoff of 50 bp were filtered. The adapter-free reads were further filtered based on the quality using the prinseq [31]. The parameters for quality trimming were set for a minimum mean quality of Q20 across the read and to trim low-quality bases at 3’ end. The minimum read length of 100 bp for the FLX reads, and 50 bp for HiSeq reads was used as cutoffs for length filtering. For the FLX reads with homopolymer sequences were trimmed using a Perl script from the NGSQC toolkit [29]. The reads corresponding to rRNA sequences were filtered using Ribopicker Perl script [32] using a plant rRNA sequence dataset generated from the rDNA sequences retrieved from NCBI (http://www.ncbi.nlm.nih.gov), TAIR (https://www.arabidopsis.org) and the rice genome annotation database (http://rice.plantbiology.msu.edu).

### *De novo* assembly of the transcriptome

All the assembling work was done on a server with 24 cores and 128 GB RAM or 64 cores and 512 GB RAM. The clean reads obtained from the 454 sequencing were assembled using the Newbler program v2.6 from Roche, TGICL v2.1 (http://sourceforge.net/projects/tgicl/) [33] and MIRA v3.9.17 (http://mira-assembler.sourceforge.net) [34]. The assembly with Newbler was carried out with six different overlap percentages of identity, i.e., 95–100% keeping the number of bases in overlap constant as 80bp, and a read was only assigned to one contig. TGICL and MIRA assemblies were done using the 98% identity over a stretch of 80 bp and keeping the rest of the parameters default.

The contigs and singletons from the Newbler 98% identity assembly were used to assemble with the 35,042 ESTs from 26 CS root-only libraries deposited in DFCI gene index, NCBI EST database (http://www.ncbi.nlm.nih.gov/genbank/dbest/dbest_access/) and Komugi wheat EST database (http://www.shigen.nig.ac.jp/wheat/komugi/ests/tissueBrowse.jsp). The hybrid assembly was carried out using the CAP3 assembly program [35] with a 98% identity across a minimum of 80-bp overlap.

The purged HiSeq reads were assembled using Velvet/Oases program version 1.0.14 (http://www.ebi.ac.uk/~zerbino/velvet/) [36] with *k-*mer values 31, 41, 51, 61, 71, 81 to get a better assembly [37]. The contigs from all the *k-*mer assemblies were clustered using CD-HIT-EST [38] at 99% identity (–c 0.95 –n 8 –T 0 –M 0 –gap -2) to remove redundant contigs generated by different *k*-mers. To further extend the contigs, the non-redundant contigs from these multiple *k-*mer assemblies were assembled into a Illumina super assembly using TGICL program [33] with 99% identity and 100-bp overlap.

The overlap between the 454 Newbler (98% identity) and the Illumina super assembly was determined by mapping the illumina reads to the 454 Newber contigs and the singletons. The reads were mapped with the mapping tool in CLCbio Genomics Workbench using the parameters same as above.

### Evaluation of assemblies

Both the FLX and the HiSeq reads used for the assemblies were mapped onto the corresponding assembled sequences using the CLCbio’s proprietary tools based on a modified version of maximal exact match approach [39] (http://resources.qiagenbioinformatics.com/white-papers/White_paper_on_CLC_read_mapper.pdf). For mapping we used the percent identity match of 95% and the length fraction of 1.0 with a global alignment.h The quality of the assembly was evaluated by aligning the assembled contigs to the full-length (FL) cDNA sequences of wheat from TriFLDB database (Riken, Japan). The FL-cDNA sequences were downloaded from TriFLDB, and redundant sequences with an identity of 99% were removed using the CD-HIT program [38]. Eventually, 17,094 non-redundant cDNA sequences were used for evaluating the completeness of our assembly. For evaluating the completeness of both the Newbler and Velvet assemblies, the program CEGMA was run on both the assemblies to determine the percent of the conserved core eukaryotic genes were assembled [40]. The HiSeq reads were also mapped to the Newbler 98% identity assembly using the mapping tool in the CLC Bio Genomic Workbench with the same parameters as indicated above. If less than five HiSeq reads were mapped in an FLX sequence, the FLX sequence will be considered unique and not present in the HiSeq sequences.

### Prediction of open reading frames and coding potential

The root assemblies were aligned with eight proteomes from finished genomes, wheat protein sequences from TriFLDB, and barley protein sequences from TriFLDB and MIPS (http://pgsb.helmholtz-muenchen.de/plant/barley) databases using the BLASTx algorithm. The sequences of the finished plant genomes, including those of Arabidopsis, rice, Brachypodium, sorghum, foxtail millet, maize, and switchgrass were retrieved from the Phytozome database (v11.0; https://phytozome.jgi.doe.gov/pz/portal.html). The BLASTx results were used to predict open reading frames (ORFs) by the findorf program [41]. A second prediction was performed on the already predicted sequences by masking the first ORF to identify the misassembled transcripts that may arise during the *de novo* assembly. TransDecoder (https://github.com/TransDecoder/TransDecoder) was used to predict ORF from the leftover transcripts. The coding potential of the transcripts without predicted ORFs were analyzed using a potential coding calculator (CPC) with default setting using a webserver (http://cpc.cbi.pku.edu.cn/).

### Functional annotation and GO assignment

The assembled transcripts were annotated by performing a BLASTx search against the NCBI non-redundant (nr) protein database with an E-value of 10E-6 and minimum coverage of 100 bp or 33 aa. Gene Ontology (GO) assignment was performed using Blast2go software (www.blast2go.com). The assembled transcripts were further aligned against the Wheat Unigene dataset build 60 (ftp://ftp.ncbi.nih.gov/repository/UniGene/Triticum_aestivum/) and against Arabidopsis, Rice and *Brachypodium* proteomes using command line BLASTx from NCBI v2.2.26 with an e-value of 10E-6. The transcripts were also searched against the Triticeae Repeat sequence database (TREP) to identify the transposable elements (TEs) in the wheat root transcriptome.

### Separating homoeologous transcripts from the *de novo* assembled transcriptome

To separate the homoeologous transcripts, we used the pipeline reported by [41] using Freebayes (https://github.com/ekg/freebayes) and Hapcut programs [42] to phase the reads based on the SNPs found in the homoeologous genes of wheat. The phased reads were assembled into contigs using a Perl script, which employs the MIRA assembler v3.4.1.1 [34] and CAP3 [35].

### Differential expression analysis in root tip and the mature root tissues

The HiSeq reads from both the root tip and maturation zone samples were mapped to the *de novo* assembled transcriptome using the read mapping tool in CLC Bio Genomic Workbench v6.5.1 (Qiagen, Carlsbad, CA, USA) with parameters set as 95% identity along the length of the read. Multiple mapping of the reads is limited to ten. The transcript abundance was calculated in terms of reads per kilobase of the transcript per million (RPKM) and transformed by adding a pseudo-count of “1” to avoid zero values in computation [43]. The transformed expression values were normalized by median scaling method across all the biological replicates of both the samples. The transcripts differentially expressed in both the tissues were identified with a fold change of at least two and a false discovery rate (FDR) *p*-value of 0.05. The normalization, statistical tests, and the p-value correction were done using the inbuilt tools in the CLC Bio Genomics Workbench. The differentially expressed genes were mapped to the MapMan bins using the Mercator tool (http://mapman.gabipd.org/web/guest/app/mercator) [44] and were represented on the metabolic pathways (http://mapman.gabipd.org/web/guest/mapman; v3.6.0RC1) [45].

## Results

### Wheat root transcriptome datasets

**Fig 1:**
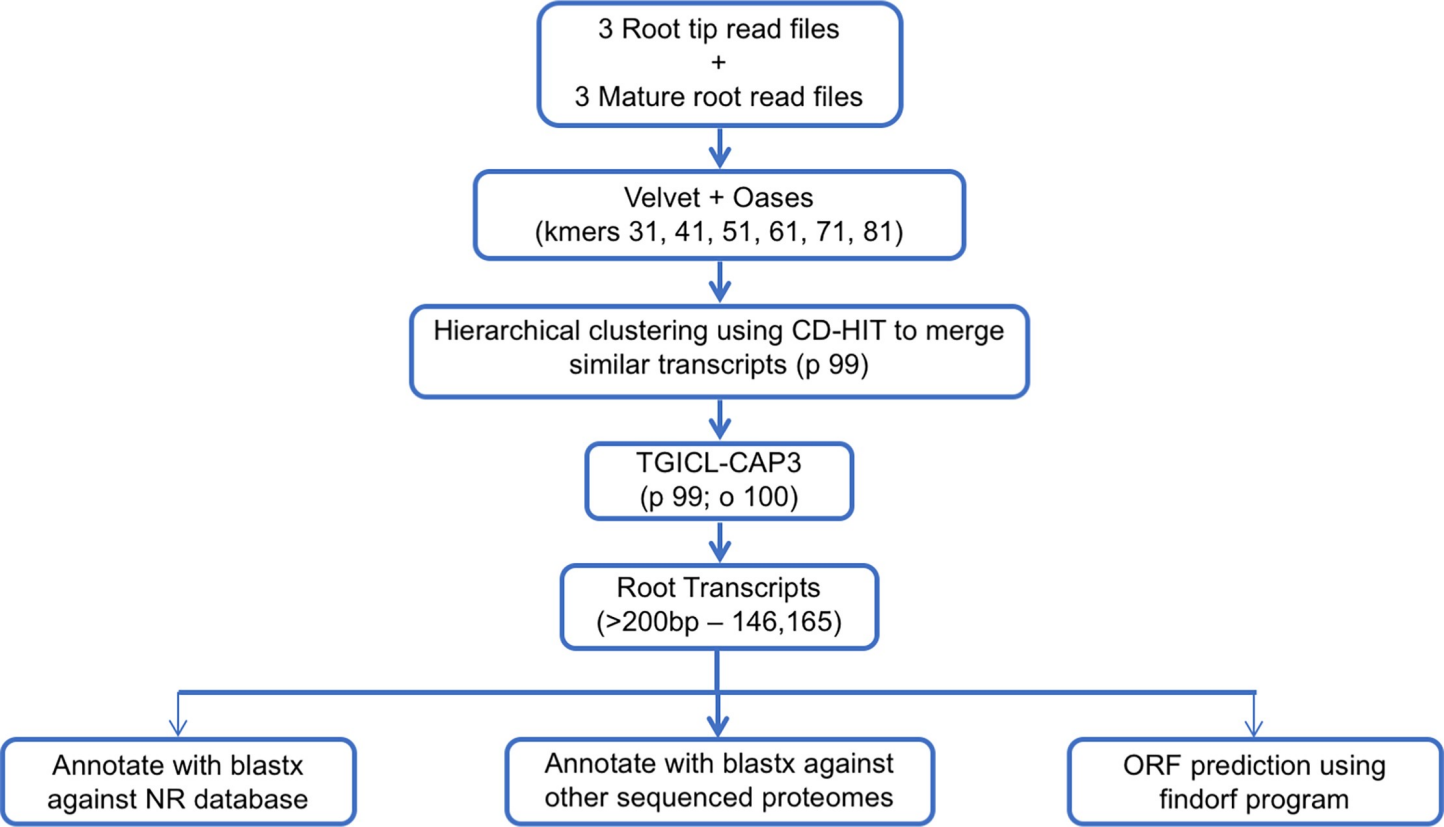
A flowchart of the assembly and annotation strategy for root transcriptome. Parameters for assemblies by Velvet/Oases, CD-HIT and TGICL-CAP3 are indicated in the parentheses.

We sequenced the transcriptome of the CS root tip using the 454 GS-FLX platform (Roche), which generated 1,086,240 raw reads from a single pyrosequencing run. As the evolution of sequencing technologies, we subsequently sequenced six libraries, three for the root tips and three from the rest of root tissues using HiSeq 2000 platform (Illumina), which generated 192,767,620 single-end sequence reads of 100 bp length. All these sequence-reads went through the processing pipeline for trimming adapters/primer sequences at the ends of the reads and low-quality bases at the 3’ end of the reads and filtering all the reads with low quality (average Phred quality score of <20) and rRNA contamination. The quality filtering and removing rRNA contamination resulted in 808,117 (74.4%) FLX reads and 169,286,239 (87.82%) HiSeq reads of high quality (Fig 1 and Table 1).

**Table 1:** Quality control and filtering of reads from 454 and Hiseq sequencing.

Sequencing runs	Total Raw Reads	Quality Filter	Contaminants	High-quality reads
454 Reads (root tips)	1,086,240	196,306	81,817	808,117 (74.4%)
Root tip Replicate 1	31,803,479	4,040,700	382,197	27,380,582 (86.09%)
Root tip Replicate 2	34,133,444	3,794,965	419,609	29,918,870 (87.65%)
Root tip Replicate 3	34,873,477	3,632,386	454,067	30,787,024 (88.28%)
Mature root replicate 1	25,348,308	2,801,923	233,118	22,313,267 (88.03%)
Mature root replicate 2	37,575,294	3,886,507	379,931	33,308,856 (88.65%)
Mature root replicate 3	29,033,618	3,166,220	293,685	25,573,713 (88.08%)

### *De novo* assembly of FLX reads and annotation of wheat root transcriptome

**Fig 2:**
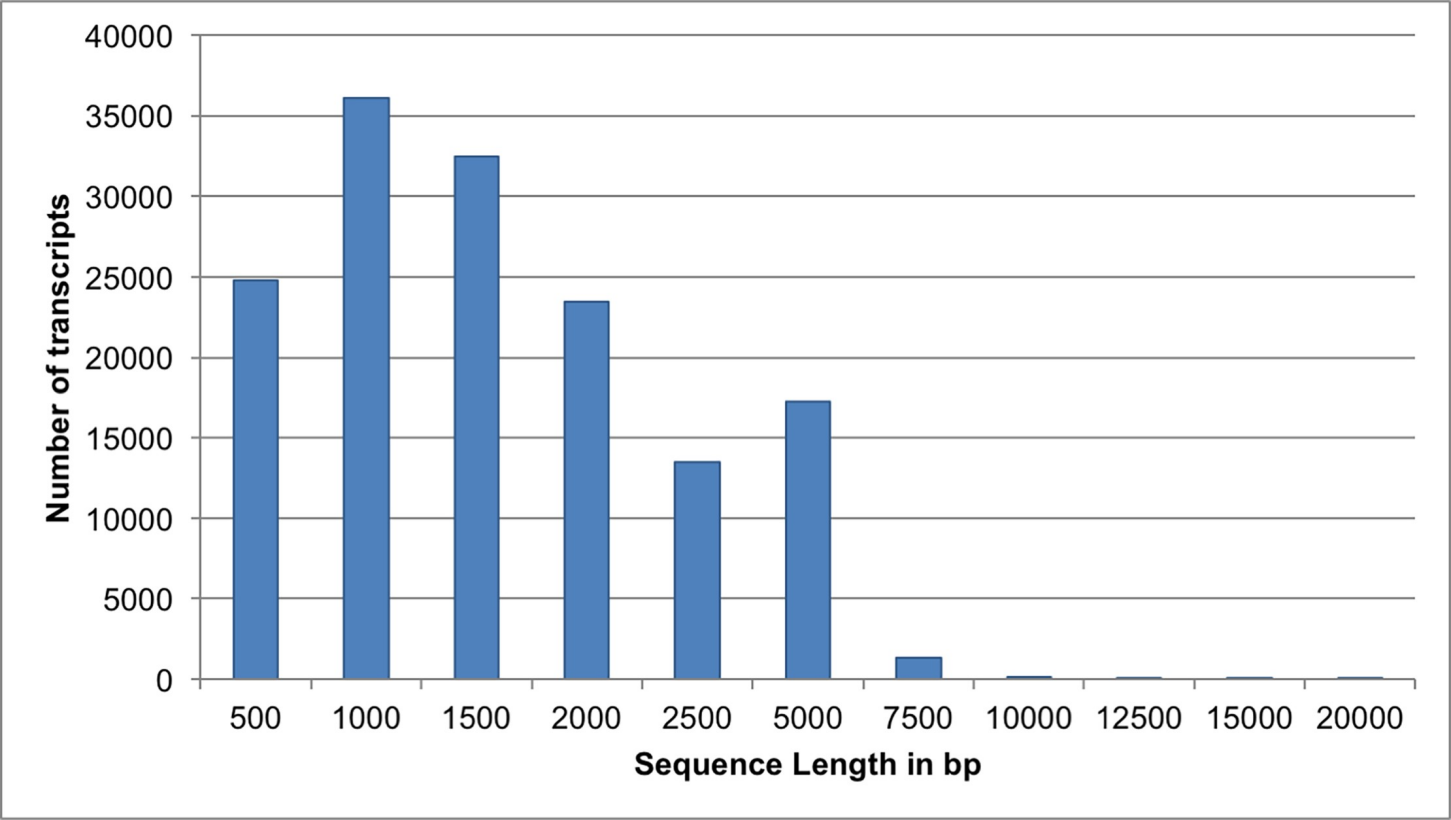
Distribution of transcript length of the Newbler assembly of the root transcriptome. Numbers in the X-axis indicate the length of the transcript in bp, and numbers in the Y-axis indicate the quantity of the transcripts.

High quality reads from 454 sequencing were *de novo* assembled using Newbler software with different identity thresholds, from 95% through 100% of identity across 80 bp overlap to place two reads into a contig. The assemblies were analyzed for various parameters, including the number of reads used, the total number of contigs generated, number of contigs longer than 200bp, N50 length, longest contig length and average contig length, and mapped the reads back onto the assembled contigs to estimate the number of unmapped reads. A total of six assemblies were generated (Table 2). As expected, an increase of sequence identity reduces N50, longest contig and average contig size, number of reads used and the size of the assemblies, but increases the number of contigs and singletons. One exception to this is the largest contig length for the assembly with 97% identity, which is smaller than that of the assembly with the identity of 98%. The 454 FLX reads were also assembled with other programs including MIRA and TGICL separately, and the quality of these assemblies was analyzed using the same output parameters used for the Newbler assemblies (Table 3). The TGICL assembly generated more contigs (78,413) than any of the assemblies from Newbler or the MIRA. But the largest contig assembled and N50 was the smallest compared to the other assemblies. The assemblies generated by TGICL and MIRA are larger (50.3 and 52.43 Mbp) than the six assemblies generated by the Newbler (Table 3). Although the Newbler assembly with 95% identity has the largest contig size and N50, use of a lower identity would increase the probability of merging the homoeologous transcripts as the homeologs from sub-genomes of wheat are known to have high similarity over 97% identity in coding sequences [40]. With all the parameters considered, the Newbler assembly with the 98% identity is overall desirable (Table 2) and used for further analysis. The distribution of the size of transcripts assembled in this assembly was shown in Fig 2.

**Table 2:** *De novo* assemblies of the FLX reads using Newbler, TGICL and Mira.

Assembly parameters	95% identity	96% identity	97% identity	98% identity	99% identity	100% identity	TGICL	Mira
Total contigs (>200bp)	23,418	23,497	24,140	24,986	25,548	22,544	78,413	73,084
Avg contig size	749.87	742.22	722.04	696.92	675.54	660.86	641.53	717.46
N50 (bp)	905	893	857	815	787	737	672	762
Large Contigs >500bp	14,720	14,640	14,866	15,122	15,400	14,314	52,763	53,271
% Large Contigs	62.86	62.31	61.58	60.52	60.28	63.49	67.29	72.89
Large contigs N50	1,046	1,033	994	951	912	835	747	828
Largest Contig size (bp)	7,528	6,892	5,977	6,699	5,598	3,787	3,451	5,199
Assembly size (bp)	17,560,564	17,439,938	17,429,939	17,413,219	17,258,803	14,898,493	50,304,196	52,434,582
Reads used in assembly	697,311	691,259	681,502	662,618	624,961	518,627	686,622	506,290
% Assembled reads	86.31	85.56	84.35	82.02	77.35	64.19	84.99	62.67
Singletons	95,262	100,789	109,525	125,932	159,474	234,805	121,295	110,157
% Singletons	11.79	12.48	13.56	15.59	19.74	29.06	15.01	13.63

**Table 3:** Assembly statistics for the Newbler, TGICL and Mira assemblies with 98% identity.

Parameters	Newbler	TGICL	Mira
Total Sequences (contigs+singletons) (>200bp)	122,086	181,533	155,628
Avg contig size	443.45	487.93	530.41
N50 (bp)	450	493	546
Largest Contig size (bp)	6,699	3,451	5,199
Large Contigs >500bp	22,047	58,467	57,927
% Large Contigs	18.06	32.21	37.22
Large contigs N50	810	725	808
Assembly size (Mbp)	54.14	88.58	82.55

To improve our assembly of the root transcriptome generated from the 454 FLX reads, we performed a hybrid assembly using the 24,986 contigs from the Newbler assembly with 98% identity (Table 2) and 35,042 ESTs from the CS root. This merged 5,863 Newbler contigs with 11,940 ESTs into 4,812 CAP3 contigs. As a result, hybrid assembly reduced the contig number from 24,986 to 23,935 and increased N50 from 815 to 887 and the longest contig size improved from 6699 bp to 6,747 bp (Table 4). At the same time, 19,123 Newbler contigs and also 23,102 ESTs found no match (Table 4), indicating that 454 sequencing expanded CS root transcriptome significantly, but its coverage is still low. This low coverage is confirmed by the CEGMA assay, which showed the root transcriptome assembled from the 454 FLX reads has a completeness of 54.84% for full length conserved eukaryotic genes (CEGs) and 85.08% for the partial CEGs.

**Table 4:** Hybrid assembly details.

Input:	
Newbler contigs	30,047
454 singletons	125,932
Sanger ESTs	35,042
Output:	
Assembly size (>200bp)	49.45 Mbp
Total CAP3 contigs	43,109
Extended Newbler or new contigs	24,149
Newbler only contigs	18,960
454 singletons	58,020
N50	489 bp
Average contig size	490 bp
Largest contig size	6,747 bp

Approximately, 87% of the transcripts had BLASTx hits in NCBI nr protein database, of which 78% of the total transcripts were assigned with GO terms and 18% were assigned with enzyme commission (EC) annotation. For biological processes, >70% of GO items fall in top five categories, i.e., organic substance metabolic process (7,227), primary metabolic process (7,224), cellular metabolic process (5,388); biosynthetic process (3,458) and nitrogen compound metabolic process (2,852). For molecular functions, the top five categories account for >80% of the total GO items, i.e., heterocyclic compound binding (5,726), organic cyclic compound binding (5,726), small molecule binding (3,727), transferase activity (3,269) and hydrolase activity (3177). For cellular localization, >90% of the GO items were from the top three groups: intracellular (11,533), membrane-bounded organelle (6,923) and membrane (3,686) localization (S1 Fig and S1 Table).

The cleaned HiSeq reads from the six libraries were mapped to the Newbler contigs and the singletons using the CLC mapping tool, and 10,277 transcripts from the 454 FLX sequencing, including 12 Newbler assembled contigs and 10,265 singletons, did not have a match with the HiSeq sequences. BLASTn of these 454 FLX sequences, that were not present in the illumina data, against the wheat genome showed that 5,115 sequences had a hit in the gene models, and search against the genome sequences showed only 413 of the Newbler transcripts were not present in the current draft genome. Search against the 5x shotgun genome [6] showed that only 169 of 413 sequences were not found. This result indicated that the 87% of the root transcriptome sequenced by 454 FLX platform has overlapped with root transcriptome sequenced by HiSeq. The rest of the 454 FLX transcriptome, though not represented in the Hiseq transcriptome, has a significant match with the wheat genome sequences. These findings show that the 454 transcriptome assembly and the Hiseq transcriptome assembly together provide a better representation of the wheat root transcriptome.

### *De novo* assembly of HiSeq sequence reads

We *de novo* assembled the clean reads that were obtained from the Illumina Hiseq sequencing using the velvet program, which assembles short reads using the De Bruijn graph, with six different *k*-mers. Multiple *k*-mer assemblies generated a total of 1,372,996 sequence contigs. Contig files from all the assemblies with *k*-mer lengths of 31, 41, 51, 61, 71 and 81 were concatenated, and the redundant contigs generated by different *k*-mer assemblies were clustered into the corresponding longest contigs using CD-HIT-EST. The concatenation resulted in 504,839 non-redundant sequences. These sequences were further assembled again using TGICL program with an identity of 99% across a minimum overlap of 100 bp to extend the contigs and generated a final super assembly of 148,984 transcripts, including 68,589 extended and merged contigs and 80,395 unextended sequences. After filtering the transcripts with a length of less than 200 bp, a total of 146,165 transcripts were assembled from the 169,282,312 quality HiSeq reads. We evaluated this assembly for various features. It has an N50 of 1,865 bp with the largest transcript of 21,400 bp and an assembly size of 210,848,484 bp. A run of the CEGMA program indicated that the root transcriptome assembled from the HiSeq reads had a completeness of 90.32% for full-length CEGs and 92.4% for the partial CEGs. This super assembly is used for the further analysis.

### Anatomy of wheat root transcriptome

**Fig 3:**
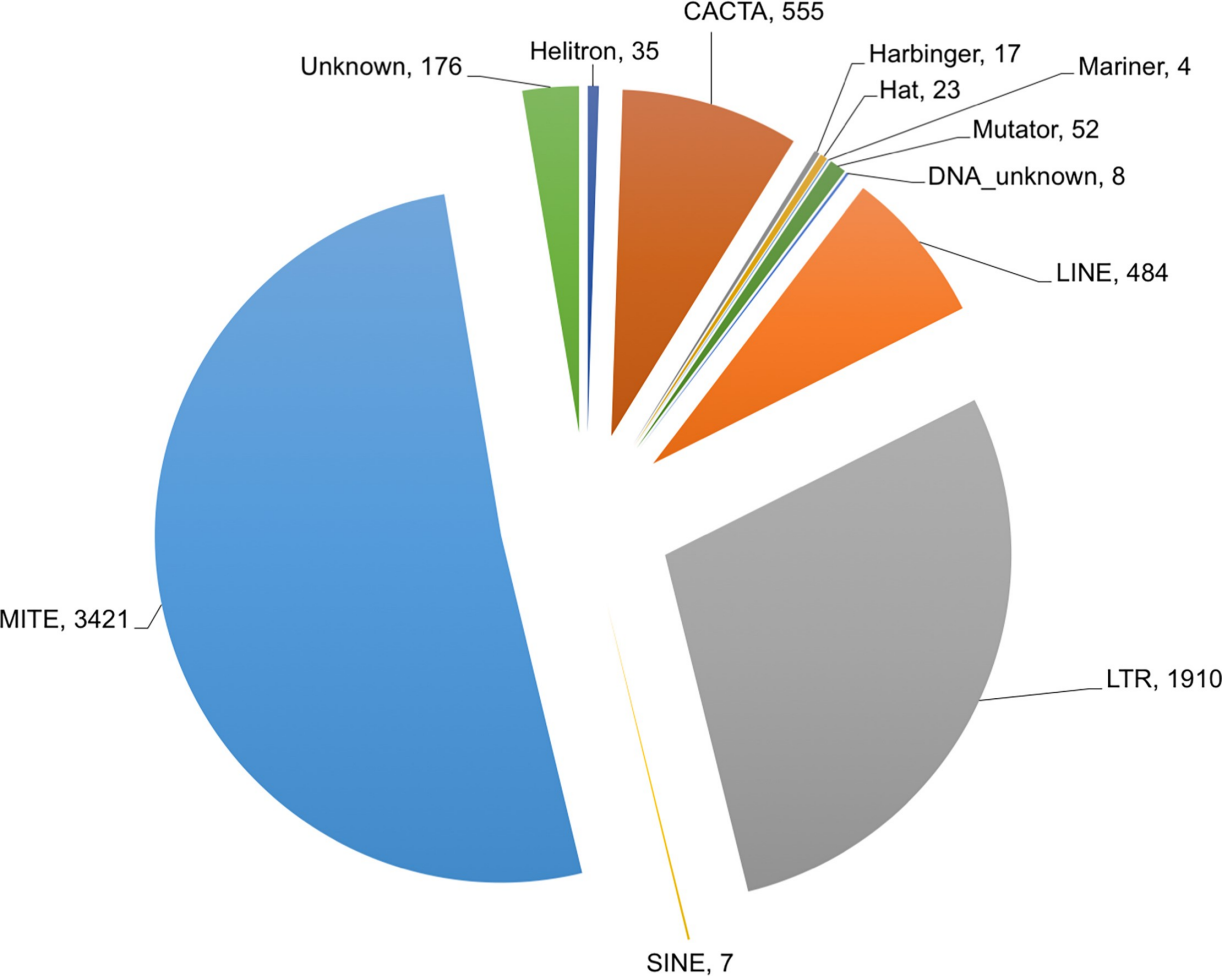
Transposable elements expressed in root transcriptome. The pie chart presents the different classes of the transposable elements expressed in the root tissues. The numbers after the transposon class are the number of transcripts in each class expressed in the root transcriptome. DNA_unknown, unknown DNA transposons; MITE, miniature inverted-repeat transposable elements; LINE, long interspersed elements; and SINE, short interspersed elements.

We analyzed the 146,165 transcripts of the wheat root transcriptome assembly developed from the HiSeq reads by aligning with public databases to classify them regarding TE-origin and coding capacity (Table 5). The HiSeq assembly was chosen due greater completeness as compared to the FLX assembly. Alignment against the Triticeae Repeat database found that 6,692 assembled transcripts originated from or containing repetitive DNA sequences were expressed in the root. These repeated sequences include 3,421 miniature inverted transposable elements (MITEs), 2,401 retrotransposons, 659 DNA transposons, 35 Helitron and 176 transposable elements of unknown classes (Fig 3). Also, 495 transcripts were found to contain repetitive sequences with transcript coverage of 90% or more, including LTRs, LINES, CACTA, Helitron and unknown classes of transposable elements. Compared to other TE species, MITEs are much smaller in size and mainly located in 3’ untranslated regions (UTRs).

**Table 5:** Anatomy of the wheat root transcriptome.

Categories		Number of transcripts	
TE-derived transcripts			3,271
Non-TE transcripts			142,894
	Pseudogenes	26,971	
	Multiple ORF-containing transcripts	8,306	
	Protein-coding transcripts	91,543	
	Non-ORF transcripts	16,074	
Total transcripts (>200 bp)			145,165

To predict the ORFs in the 142,894 non-TE transcripts, we performed a BLASTx run against various protein databases, and the BLASTx outputs were used with the findorf program to predict the coding sequences (cds) and protein sequences encoded by the transcripts. The program predicted ORFs in 116,833 transcript sequences, and the remaining 26,061 sequences had no coding capacity. Of the 116,833 ORF-containing transcripts, 4,727 sequences had premature stop codons, and 18,000 sequences had frame-shifts in their ORFs, suggesting that these 22,727 sequences were transcribed from pseudogenes. For the 94,106 transcripts that contain normal ORFs, running an iterative step of findorf with the first ORF masked found that 6,158 sequences contained a second ORF, suggesting that they were derived from misassemblies during the *de novo* assembly process. Therefore, a total of 87,948 transcripts contain unique ORFs. Further annotation of the 26,061 transcripts, from which no ORFs were predicted by findorf, using outputs of BLASTx against NCBI nr database predicted ORFs in 9,987 transcripts. Of these 9,987 transcripts, 4,244 transcripts were found to be pseudogenes with a frameshift or a premature stop codon in the ORF. And an iterative run with the masked ORF sequence found 2,148 transcripts were containing a second ORF. Thus, 3,595 transcripts were identified with a functional ORF, increasing the total transcripts with a predicted functional ORF to 91,543. These transcripts were considered high-confidence (HC) protein-coding transcripts.

The findorf program did not predict any ORF in the remaining 16,074 transcripts. Using TransDecoder (https://github.com/TransDecoder/TransDecoder), we identified only a single putatively functional ORFs in 2,404 of these 16,074 transcript sequences based on the pfam domain and the BLASTP hit against the SWISSPROT database. These 2,404 transcripts are therefore considered low-confidence (LC) protein-coding transcripts.

The remaining 13,181 transcripts were left over without any predicted ORF present and further analyzed using the potential coding calculator (CPC). Of the 13,181 transcripts, 189 showed coding potential with the score ranging from 3.999 to 0.008, 12,705 showed no coding potential with a potential coding score ranging from -0.008 to -1.572, and 287 transcripts had no results returned by CPC. Considering that LC proteins are not confirmed in other plant genomes and due to the very low CPC for the noncoding transcripts, they were pooled and referred as non-ORF transcripts hereafter.

We aligned the 91,543 ORF-containing transcripts and 16,074 non-ORF transcripts with the current version of the wheat genome assembly [7]. The results showed that 58,341 (63.7%) ORF-transcripts found matches in whole genome sequence with >97% identity and >50% length coverage. A majority (51,610) of these ORF-transcripts had hits in the predicted gene models (S2 Fig), 6,252 ORF-transcripts did not show any sequence similarity to the predicted cDNA sequences, and 536 showed no homology to the wheat genome assembly. Of the 16,074 non-ORF transcripts, 10,931 hit the whole genome sequences with the above criteria, and 360 did not show any match in the wheat genome assembly. Of the 10,931 matched non-ORF transcripts, 2,343 hit the predicted cDNA sequences with same parameters and remaining 8,588 only found matches in the wheat genome assembly but not in the predicted cDNA, suggesting that they are located either in the intergenic regions or introns. To further validate the 536 ORF-containing transcripts and 360 non-ORF transcripts that are not found in the IWGSC (International Wheat Genome Sequencing Consortium) draft genome and gene models [7], we did a BLASTn search of these sequences against the 5x wheat genome sequences assembled using 454 sequencing platform [6]. Only 20 HC protein-coding transcripts and 43 non-ORF transcripts were not found. These results indicate that almost all the ORF-containing and non-ORF transcripts are present in the wheat genome, but the current wheat genome assembly and annotation is incomplete.

To gain insights into the organ specificity of the transcripts, we aligned the wheat root transcriptome assembly with the RNA-Seq reads from the aboveground tissues, i.e., leaf, stem, spike, and grain, of wheat plants, which are deposited in NCBI SRA database. Results showed that 6,222 (6.8%) of the 91,543 protein-coding transcripts and 834 (5.2%) of the 16,074 non-coding transcripts did not show significant similarity, indicating that they are root specific.

Common wheat is a hexaploid species containing the A, B, and D genomes. During the *de novo* assembly of the reads into transcripts, the reads corresponding to the homoeologous genes can be merged into a single transcript rather than into separate transcripts due to high sequence similarity between the homoeologous genes [6, 7]. In our assembly pipeline, we merged multiple *k*-mer assemblies, which reduced the redundancy in the assembled contigs. This strategy also merged homoeologs with high sequence similarity into one contig. With available assembly algorithms and *de novo* assembly programs, however, it is difficult to assemble highly similar sequences into separate contigs. Using the homoeolog separation pipeline [41], we identified a total of 13,664,029 polymorphic reads corresponding to the 34,506 of the 91,543 assembled transcripts with a predicted functional ORF. These reads were assembled into 115,692 homoeologous blocks using the phasing information provided by the hapcut program.

**Fig 4:**
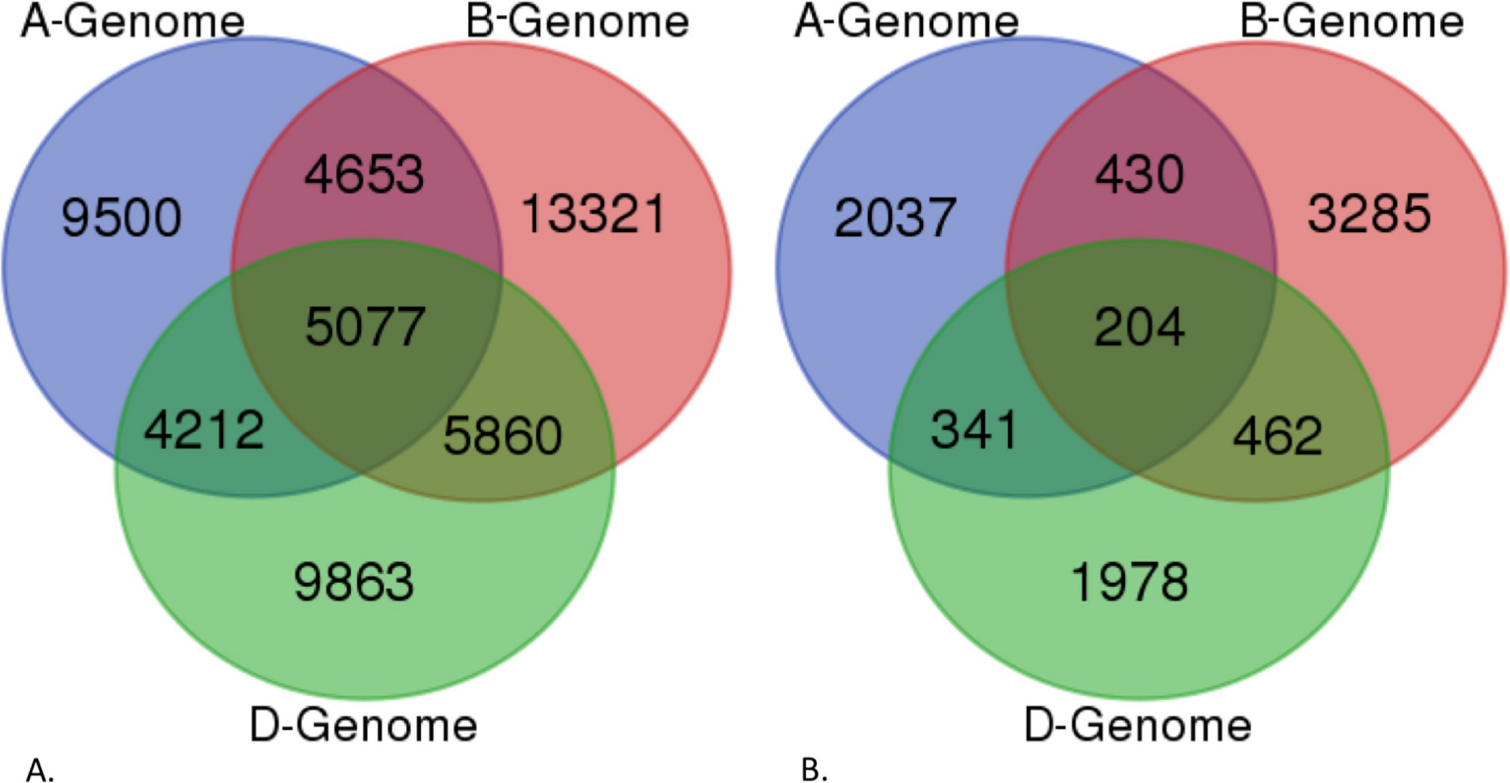
Venn diagram showing the distribution of protein-coding and non-ORF transcript nucleotide sequence alignment with the IWGSC draft genome sequences separated into sub-genomes. A) Distribution of the alignment of protein-coding transcripts with the sub-genome separated chromosome sequences. B) Distribution of the alignment of non-ORF transcripts with the sub-genome separated chromosome sequences. The sub-genomes A, B, and D are represented by color, and the numbers within the circles indicate the number of transcripts aligned in each of the sub-genomes.

**Fig 5:**
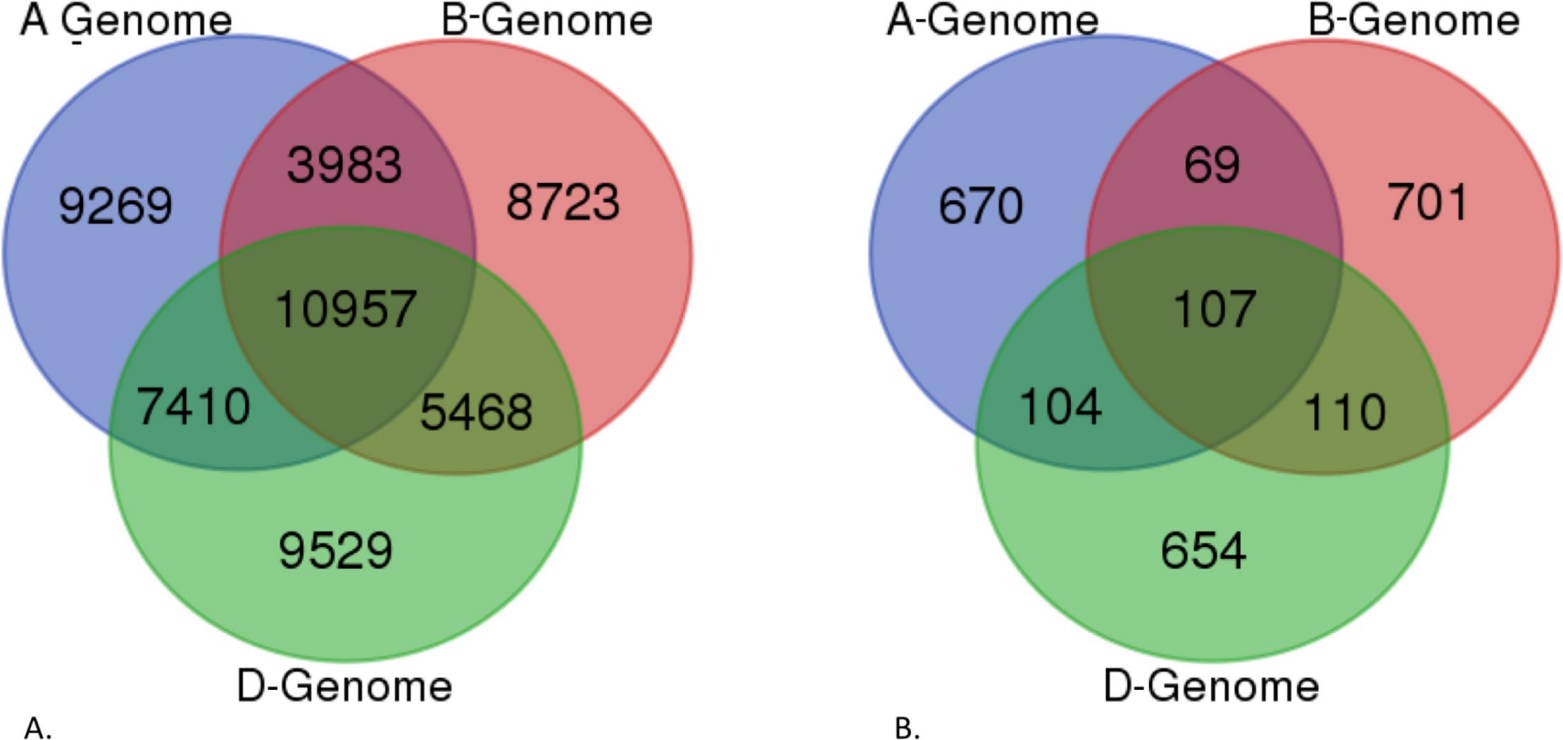
Venn diagram showing the distribution of protein-coding and non-ORF transcript nucleotide sequence alignment with the IWGSC cDNA sequences separated into sub-genomes. A) Distribution of the alignment of protein-coding transcripts with the sub-genome separated cDNA sequences. B) Distribution of the alignment of non-ORF transcripts with the sub-genome separated cDNA sequences. The sub-genomes A, B, and D are represented by color, and the numbers within the circles indicate the number of transcripts aligned in each of the sub-genomes.

To gain an understanding of the sub-genome specific expression of the assembled root transcriptome, we pooled the chromosomes and the gene models in the draft genome into subsets of the A, B, and D genomes and aligned the ORF-transcripts and the non-ORF transcrits with them using the same parameters as above (Figs 4 and 5). The results showed that 52,486 ORF-transcripts and 8,737 non-ORF transcripts had a hit in the genome and that 55,704 ORF-transcripts and 2,415 non-ORF transcripts had a hit and the cDNA. All these corroborate that the current assembly and annotation is incomplete for each sub-genome.

In the 5,115 transcripts from the 454 assembly that were absent in the HiSeq assembly but have a hit against the gene models from the wheat draft genome sequence, 2,203 transcripts share a hit in the wheat gene models with a HiSeq transcript. These could be fragments of the same gene or a homeolog. Only ten transcripts were the contigs, and the remaining 2,193 were singletons. This indicated that these reads could have been generated from the low expression genes resulting in a very low representation. The remaining 2,912 transcripts were predicted for the ORFs using the findorf program, which identified 1,904 transcripts with an ORF. Of these, 740 and 282 have frameshit and a premature stop codon, respectively, and 882 with an ORF coding for a functional protein. Of the transcripts with a functional ORF, four transcripts were the contigs generated by the newbler and the remaining 878 were the singletons. Thus, the root transcriptome assembly contains 92,335 ORF-containing transcripts, 91,453 from the HiSeq reeds and 882 from the FLX reads.

### Functional annotation, classification, and comparative genomics

**Fig 6:**
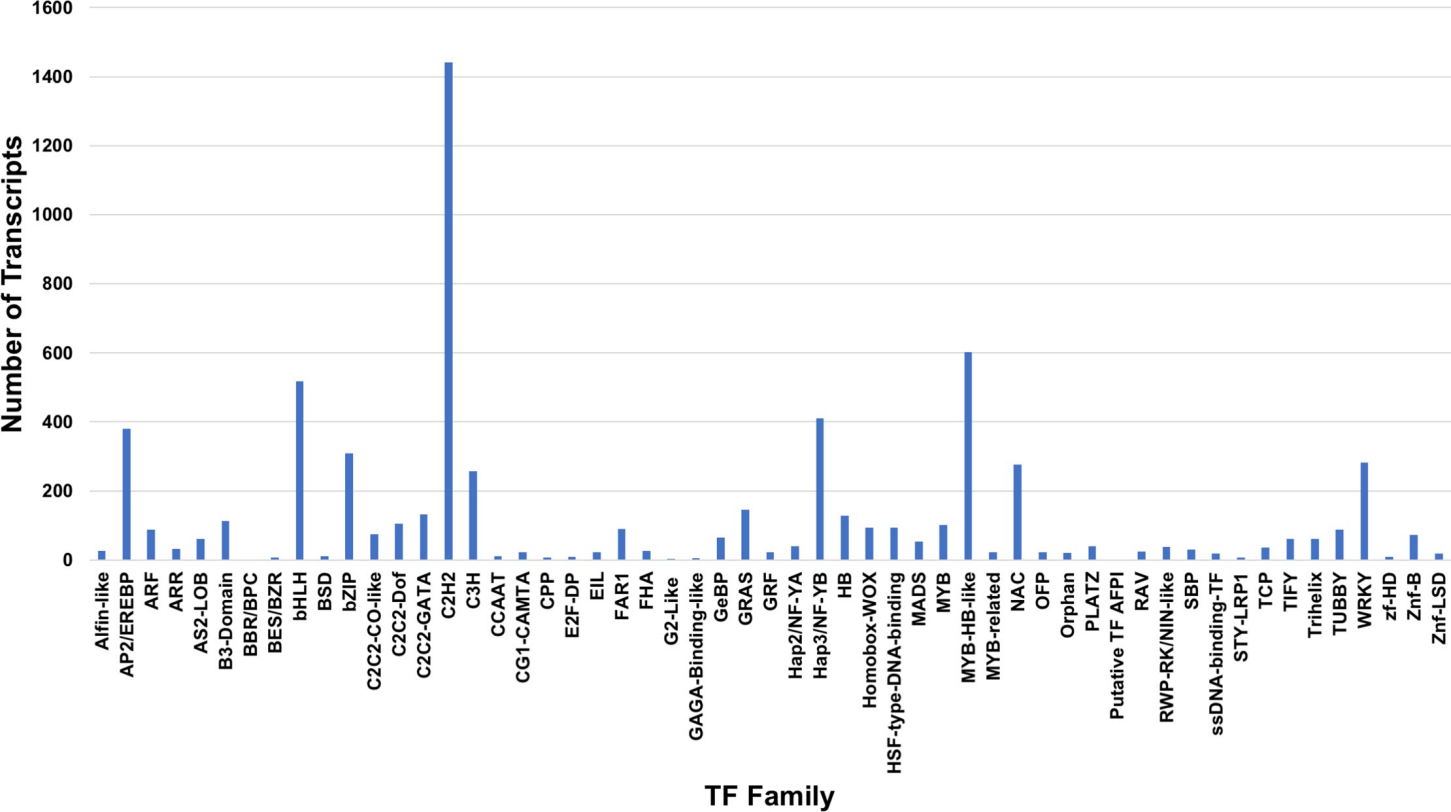
Transcription factor (TF) families expressed in both the root tissues used in the study. Numbers of transcripts in each TF family are indicated on the X-axis, and names of the TF families are indicated on the Y-axis.

The assembled transcripts were annotated by aligning against the NCBI nr protein database. Out of the 92,335 *de novo* assembled transcripts predicted with a functional ORF, which were combined from the HiSeq assembly and the FLX assembly,86,477 (94.47%) transcripts have at least one hit in the nr database, and 5,066 (5.53%) transcripts with a predicted ORF don’t have a hit in the database. GO terms were assigned based on the annotation of the nr database, and 71,031 (77.59%) transcripts were assigned to at least one GO term. For 15,446 (16.87%) transcripts, there is a hit in the nr database, but no GO term is assigned. For biological processes, the top five GO groups account for >75% of the GO-assigned transcripts. These include macromolecule metabolic (13,828), organic cyclic compound metabolic (10,080), cellular aromatic compound metabolic (10,072), heterocycle metabolic (10,054) and cellular nitrogen compound metabolic process (10,047) (Fig A in S3 Fig and S2 Table). For molecular functions, top three GO groups, nucleoside phosphate binding (14,288), nucleic acid binding (9,987) and transferase activity, transferring phosphorus-containing groups (6,412) account >42% of total GO-assigned transcripts (Fig B in S3 Fig and S2 Table). The assembled root transcriptome has 6,594 transcripts coding for transcription factors (TFs) of 55 families. The C2H2 TF family is the largest with 1,442 members followed by Myb-HB-like (601), bHLH (518), HAP3/NF-YB (411) and AP2/EREBP (380) in the top five families (Fig 6 and S3 Table). For subcellular localization, 75.6% (42,785) predicted proteins are located in intracellular space, 18.4% (10,434) in cell periphery, and 3.2% (1,811) in organellar lumen (Fig C in S3 Fig and S2 Table).

**Fig 7:**
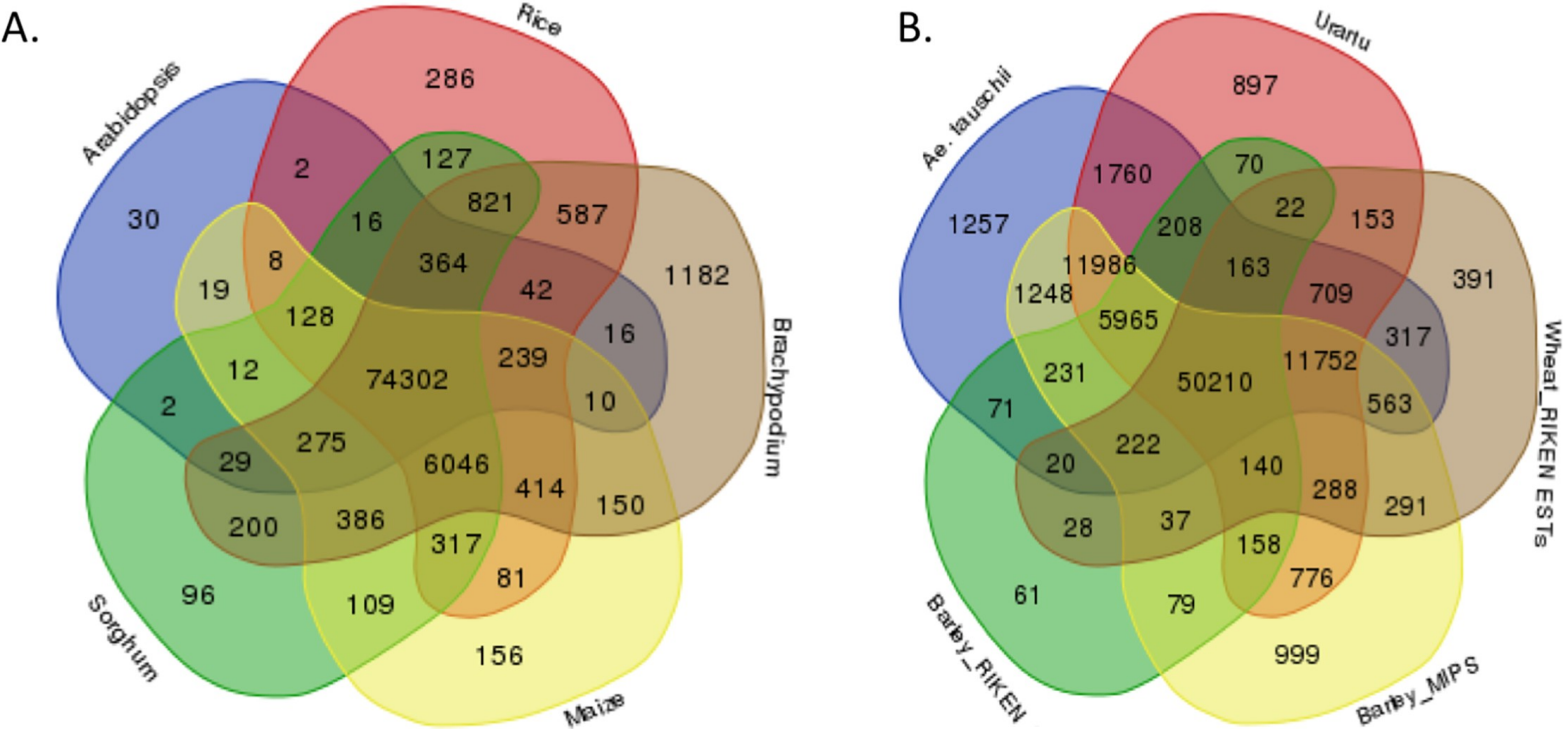
Venn diagrams showing the similarity of wheat root transcriptome with finished and draf genomes of model and crop plants. A) Comparision of the root transcripts against the protein sequences of finished genomes. B) Comparision of root transcripts against the protein sequences of draft genomes and assembled ESTs. Arabidopsis (TAIR v10); Rice (RGAP v 7); Brachypodium (Pyhtozome, Bd192); Sorghum (Phytozome, Sb79); Maize (Phytozome, Zm181); *Ae*. *Taushii* (Jia et al., 2012); Urartu (*Triticum urartu*) (Ling et al., 2013); Wheat_RIKEN_ESTs and Barley_RIKEN–translated protein sequences from assembled ESTs at RIKEN (http://igenomeinfo.riken.jp/English/database_e.html#18); Barley_MIPS–protein sequences from barley genome from MIPS (ftp://ftpmips.helmholtz-muenchen.de/plants/barley/public_data/).

To further investigate the similarity of the wheat root transcriptome with the finished and draft genomes of model plants and other crops, we aligned the root transcripts with proteins sequences from Arabidopsis, Brachypodium, rice, sorghum, maize, *Ae*. *taushii*, and *T*. *urartu* from NCBI and protein sequences for wheat and barley from RIKEN and MIPS using BLASTx. The hits from each database are compared. In the finished genomes, 74,302 (81.16%) transcripts had a match in all the genomes while 30, 96, 156, 286, 1,182 transcripts were unique to Arabidopsis, sorghum, maize, rice, and Brachypodium, respectively. Whereas in the draft genomes and ESTs, only 50,210 (54.84%) had a match owing to the incompleteness of the genomes (Fig 7).

### Differential expression analysis of root tip and the mature root tissues

The reads from the libraries corresponding to the root tip and the mature part of the root were mapped to assembled transcripts, and their abundance was quantified in these two tissues. Of the 107,617 transcripts (91,543 orf-transcripts + 16,074 non-ORF transcripts) assembled from the HiSeq reads, a total of 1,728 transcripts were found differentially expressed between the root tip and mature root tissues according to a comparison of expression levels with fold change (FC) ≥ 2 and a false discovery rate (FDR) of ≤ 0.05. Out of these 1,728, 1083 transcripts were more abundant in root tips, and 645 transcripts were more abundant in the matured part of the root. A search of the NCBI nr database and the Arabidopsis TAIR database annotated 1,647 of the 1,728 differentially expressed transcripts (DETs). Remaining 81 transcripts had no annotation in both the databases, of which 27 transcripts have functional ORFs but do not have a match in the two databases used, whereas 54 were non-ORF transcripts, representing putative noncoding transcripts. Of the 27 transcripts containing ORFs but no annotation, 18 were enriched in root tips and 9 in the mature root; and out of the 54 non-ORF transcripts, 25 were enriched in root tips and 29 in mature root tissue.

Of the 1,728 DETs in the root tips, 82 transcripts were without any predicted ORF and considered noncoding. Interestingly, 41 transcripts were up-regulated and 41 down-regulated. For 15 transcripts upregulated in root tips transdecoder predicted single putative functional ORF and for another six transcripts were predicted with more than one ORF. In the down-regulated transcripts, 11 transcripts were predicted with a single ORF, and three transcripts were predicted with more than one ORF.

**Fig 8:**
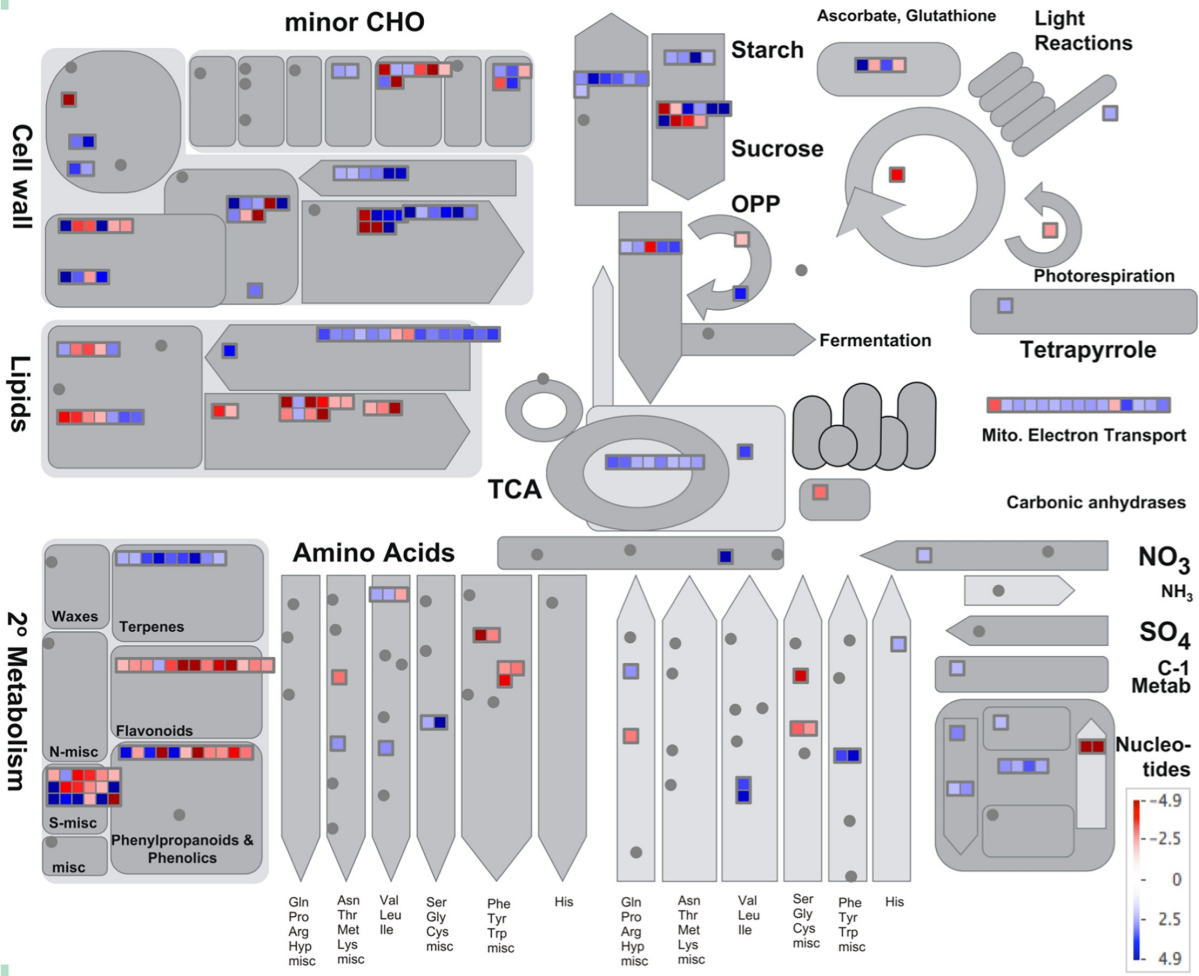
An overview of the differentially expressed transcripts mapped onto the metabolic pathways. The differentially expressed genes were mapped onto the metabolic pathways using MAPMAN software. Each box represents a transcript, and the red colored ones are the up-regulated in the mature root tissues, and the blue colored ones are induced in the root tips. A fold change scale is indicated in the lower right corner.

We annotated the DETs by BLASTx against the protein databases and mapped them onto the metabolic pathways using MapMan. Full annotation of the DETs is listed in S4 Table and an overview of the metabolic pathways in which the differentially expressed genes in root tip and mature root were illustrated in Fig 8. Genes in several metabolic pathways showed consistent differential expression, including fatty acid (FA) metabolism, secondary metabolism, glycolysis and tricarboxylic acid (TCA) cycle, cell wall biosynthesis and degradation (Fig 8). A total of 248 DETs were represented on the overview pathway map (Fig 8). Of the 248 mapped transcripts, 51 were involved in the secondary metabolism, 43 in lipid metabolism, 38 in cell wall metabolism, 23 in amino acid metabolism, 20 in starch and sucrose metabolism, 20 in minor carbohydrate metabolism, 13 in glycolysis and TCA cycle and 15 in the mitochondrial electron transport pathway.

Root tips include apical meristem, which maintains the high activity of cell division. In agreement with this, a significant number of up-regulated transcripts in the root tips were involved in the protein synthesis. These transcripts encode the ribosomal subunit proteins (93 transcripts), translation (52 transcripts), chromatin structural proteins like histone proteins (39 transcripts), RNA binding and splicing components (27 transcripts), transcription factors (25 transcripts), and transport (21 transcripts) (S4 Table). Several metabolic pathways were up-regulated in root tips: TCA cycle and mitochondrial electron transport pathways, FA synthesis, terpene synthesis, and biosynthesis of aromatic amino acids Phe, Tyr and Trp. In contrast, mature root mainly functions in cell elongation, differentiation, the formation of root hairs and lateral roots and transportation of water and minerals. Accordingly, nine genes in the phenylpropanoid pathway for lignin biosynthesis were enriched in the mature root tissue in agreement with its function in water conduction. These include those encoding a phenyl ammonia lyase (PAL), a 4-hydroxycinnamoyl CoA ligase (4CL), a hydroxycinnamoyl-Coenzyme A shikimate/quinate hydroxycinnamoyltransferase (HCT), a cinnamoyl-CoA reductase (CCR), a Caffeate O-methyltransferase (COMT) and four 4CL-like proteins. Except for the *COMT*, expression of these genes was induced in mature root tissues. Closely related to phenylpropanoid pathway, expression of the flavonoid pathway genes was also increased in the mature root. Pathways for FA degradation and biosynthesis of polar uncharged amino acids, Ser, Gly, and Cys, were also up-regulated in root tips (S4 Table).

Of the metabolites, carbohydrate metabolism regulates root development in numerous ways apart from providing energy and structural components, including gravitropism, osmotic adjustment, and sugars that often act as regulatory signals and are required for lateral root initiation. We found that 22 transcripts corresponding to the different enzymes in the starch and sucrose metabolism were differentially expressed in root tips and mature root tissues (Fig 8). Transcripts (TC039764, TC088166, and TC088167) encoding for the AGPases, starch synthases and starch branching enzymes in the starch biosynthesis pathway were induced or up-regulated in the root tips. At the same time, transcripts encoding enzymes of starch degradation, such as starch D enzyme, starch phosphorylase, and heteroglycan glucosidase were induced in the root tips, indicating active starch metabolism in the root tip tissue. In another aspect, three transcripts (TC001776, TC071737, and TC110592) encoding sucrose synthase were induced in the mature root.

Phytohormones, particularly auxin, brassinosteroids (BRs), jasmonic acid (JA) and abscisic acid (ABA), regulate almost every aspect of root development and growth. Numerous transcripts encoding hormone biosynthetic enzymes and transporters were also differentially transcribed between root tips and the mature root portion. Five auxin-promoting transcripts, one encoding the auxin efflux carrier PINFORMED 2 (PIN2), similar to OsPIN2 of rice and AtPIN7 of Arabidopsis, and four coding for an auxin-inducible 5NG4/Nodulin21-like protein (TC144456) and O-fucosyltransferases, were up-regulated in the root tip compared to the mature root. In contrast, six auxin-suppressing transcripts, three encoding Aux/IAA proteins homologous to OsIAA2, OsIAA6, and OsIAA21 of rice, and three coding for indole-3-acetic acid (IAA)-amido synthase-like proteins, which prevent free IAA accumulation, were up-regulated in the mature root. Downstream in the auxin pathway, two transcripts, TC056398 and TC018213, encoding SMALL AUXIN UPREGULATED (SAUR) proteins were differentially expressed with the former induced in the root tip and the latter induced in the mature root. Three transcripts encoding ATP binding cassette subfamily B/multi-drug-resistance/P-glycoprotein (ABCB/MDR/PGP) were up-regulated in the root tips. These proteins were identified to create the auxin gradient together with other auxin influx carriers [46]. In the BR biosynthesis pathway, three transcripts, two for cycloartenol synthases and one for the DWF1 protein, which is involved in the conversion of early brassinosteroid precursor 24-methylenecholesterol to campesterol [47], were up-regulated in the root tips. These results suggest that a higher auxin and BR level is maintained in root tips compared to the matured zone. In the JA signaling pathway, three transcripts encoding the sulfotransferases similar to AtST2A, a protein involved in the reduction of the endogenous levels of 12-OH-JA (a by-product of switching off JA signaling) [48], were up-regulated in the mature root, suggesting an opposite pattern for JA as compared to auxin and BRs. A complicated scenario was observed for the ABA biosynthetic pathway. Three transcripts homologous to Arabidopsis ABA DEFICIENT 2 (ABA2)/SHORT-CHAIN DEHYDROGENASE/REDUCTASE 1 (SDR1) and one homologous to aldehyde oxidase 2 (AAO2), a putative ABA aldehyde oxidase that may be functional in the last step of ABA biosynthesis [49], were induced in the mature root. Two transcripts coding for TETRATRICOPEPTIDE-REPEAT THIOREDOXIN-LIKE 1 (TTL1) were up-regulated in root tips. TTL1 in Arabidopsis is required for elongation and organization of the root meristem and is involved in ABA signaling [50]. Two transcripts encoding for cytokinin receptor HISTIDINE KINASE 3 were induced in the mature root.

**Fig 9:**
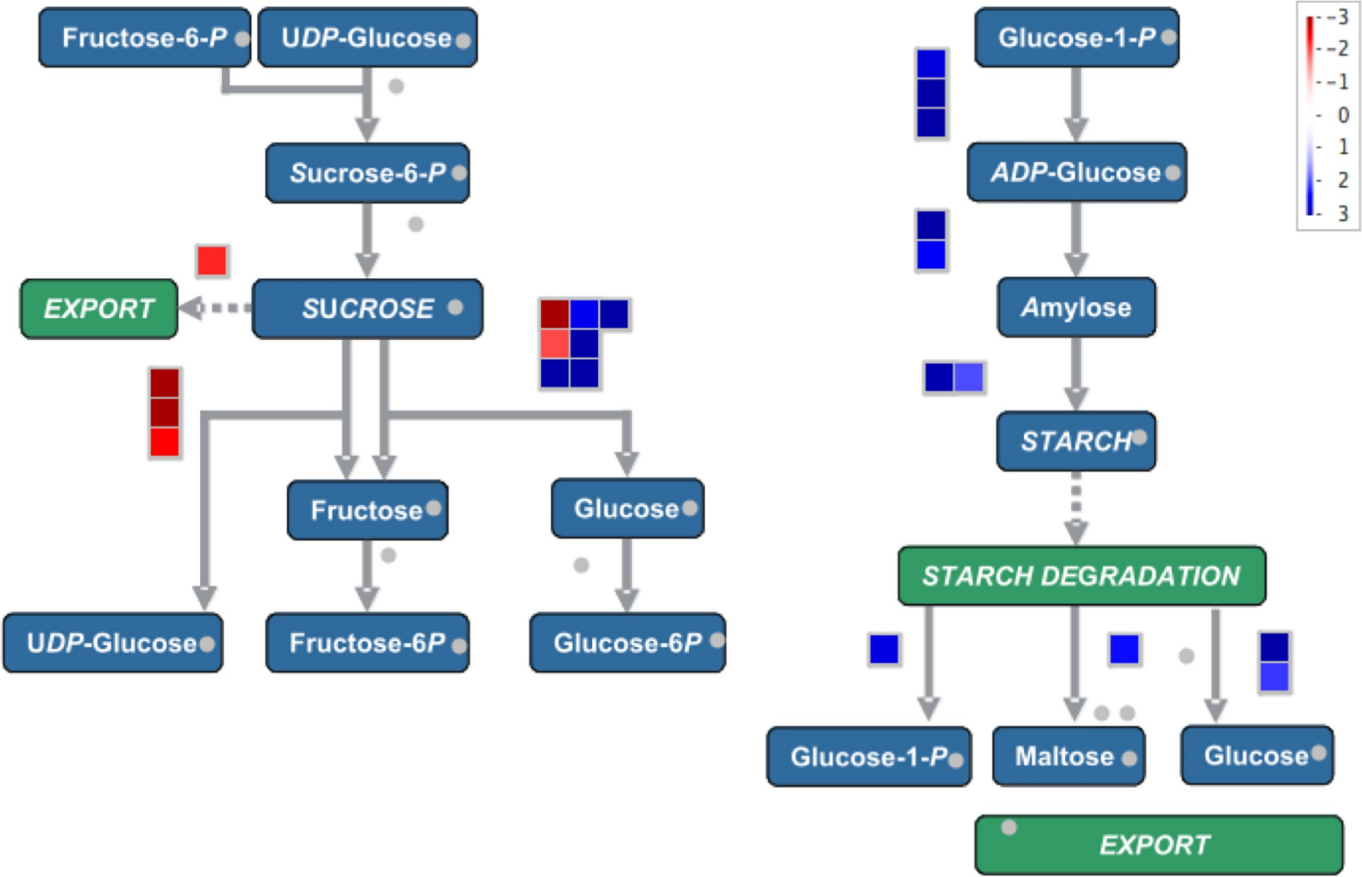
Differentially expressed genes in root tips and mature root involved in the starch biosynthesis. The transcripts encoding for the enzymes involved in the starch and sucrose metabolism were represented each by a box. The blue colored are induced in the root tips and the mature root tissue. A fold change scale is indicated in the upper right corner.

**Fig 10:**
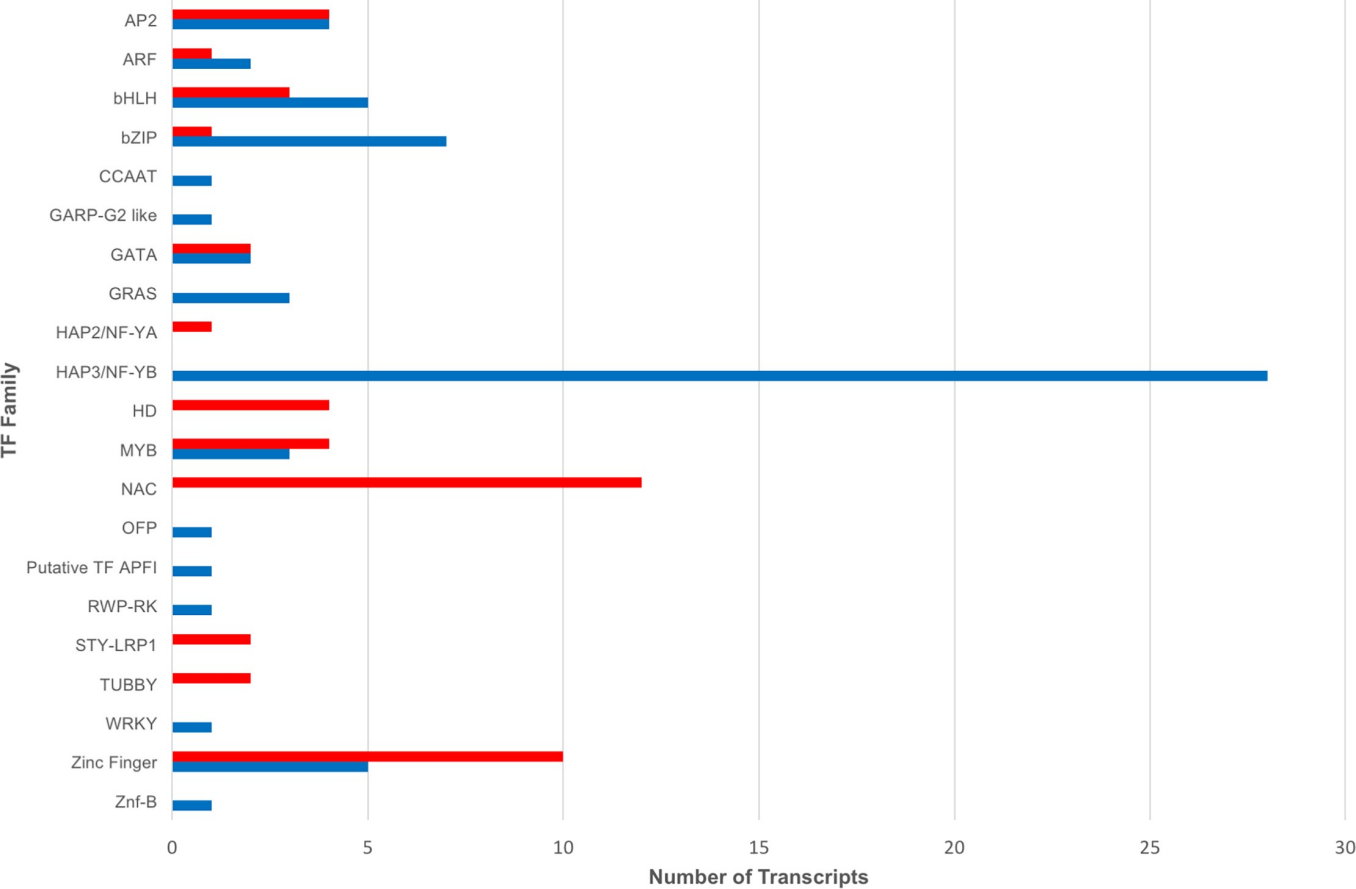
Transcription factor (TF) families differentially expressed in root tip and the mature root tissues. Numbers of transcripts in each TF family are indicated on the X-axis, and names of the TF families are indicated on the Y-axis. The striped bars are the transcripts induced in mature root and the solid black bars in the root tips.

Transcription factors (TFs) are important regulators of gene expression. Expression of 112 transcripts encoding TFs of 21 families was altered in wheat root along the longitudinal axis. The major classes include AP2, bHLH, bZIP, MYB and MYB-related, homeodomain (HD), NAC families, and numbers and expresstion patterns of these TF transcripts are shown in Fig 9. Notably, all 38 members of nine TF families, including three members of the GRAS family and 28 members of the NF-YB family, were induced in the root tips. By contrast, all 21 members of five TF families, including 12 members of the NAC family, four members of the HD family, were only induced in the mature root tissue. For the remaining seven TF families, such as the MYB family, 28 members were up-regulated, and 25 members were down-regulated in root tips (Fig 10). Several differentially expressed TFs are homologous to the known genes functioning in root development in the model plants, including two members of the STY-LRP1 family upregulated in the mature root tissue, suggesting their involvement in lateral root development. Of the four members of the AP2 family that up-regulated in root tips, three are homologous to AINTEGUMENTA-like 5 of *Ae*. *taushcii* (AIL5; EMT02119) and another homologous to BABY BOOM 2 (BBM2; EMS64473) of *T*. *urartu*. Two transcripts encoding for the ARFs homologous to AUXIN RESPONSE FACTOR 6 Arabidopsis thaliana (AtARF6) were up-regulated in root tip, and another transcript encoding for ARF homologous to AtARF11 was induced in mature root part. One transcript (TC084552) encoding the Argonaute family member homologous to AtAGO4 that is associated with 24-nt smallRNA and involved in RNA dependent DNA methylation [51] was induced in root tips.

## Discussion

Growing and functioning underground complicates root studies by using traditional approaches, leaving a gap in our understanding of wheat development and growth. Transcriptome analysis by RNA-Seq technology promises new opportunities for studying root development. RNA-Seq technology has been used to characterize the response of wheat root transcriptome to phosphate starvation [52] and infection of *Gaeumannomyces graminis* var. *tritici*, a pathogen of take-all root rot disease [53], but a reference transcriptome of wheat root and developmental expression pattern are not available. The present study developed and characterized a *de novo* assembly of wheat root transcriptome containing 94,106 transcripts that contain unique ORFs and identified 1,728 differentially expressed transcripts between the root tip and mature root tissues. All this will provide a global view of wheat root transcriptome and start point for a molecular understanding of root development and improving soil-related stress tolerance in a reverse genetics approach.

### Root transcriptome assemblies

We assembled the FLX reads into a transcriptome of 19,123 Newbler contigs with >50% completeness and the HiSeq reads into a transcriptome of 146,165 transcripts with >90% completeness. For the FLX reads, the Newbler assemblies performed better overall on the statistics metrics than TGICL and Mira. Compared to the recently reported transcriptome assemblies of wheat [19], barley [54], *Persea Americana* [55] and smooth cordgrass [56], our Newbler assembly showed comparable or even better statistic metrics including N50 value and percentage of assembled reads. Compared to the Newbler assembly of the pyrosequencing reads, the assembly of the HiSeq reads had a much greater N50 value, assembly size, and completeness mainly due to the large read number. A total of 1,749 transcripts from the HiSeq assembly found matches in the wheat genome sequences but did not get hits in the publicly available RNA-Seq reads from the wheat roots. This discrepancy is mainly due to the enrichment of them in root tips by separation of root tips from the rest of the root in the present study. All these corroborate sound quality and high content of information of the HiSeq assembly of the wheat root transcriptome.

Common wheat is a hexaploid species with A, B, and D genomes and a total of 94,000 to 120,000 protein-coding genes [6, 7]. Of the 91,543 transcripts, 34,506 were separated into 115,692 homoeologous blocks. If each of these 34,506 transcripts was derived from merging of at least two homoeologous transcripts, the total number of transcripts in the root assembly would be >126,049, excessing the total gene number, implying the existence of isoforms of transcripts due to alternative splicing, which is enhanced in polyploid wheat [57]. In another aspect, 6.8% protein-coding transcripts did not find a match in the current assembly of the wheat genome, indicating the incompleteness of wheat genome assembly. In these respects, the wheat root transcriptome assemblies from this research can be used for improving wheat genome assembly and annotation.

Of the 146,165 transcripts in the final assembly of the HiSeq reads, 91,543 transcripts contain predicted functional ORFs, 26,971 transcriptes were transcribed from pseudogenes, and 13,181 transcripts have no coding capacity and do not show homology to degenerated TEs, suggesting that they were transcribed as polyadenylated long non-coding RNAs (lncRNAs) (Table 5). The high percentage of pseudogenic transcripts in the wheat root transcriptome is consistent with the discovery that of the 7,264 predicted protein-coding genes on wheat chromosome 3B, 1,938 (26.7%) are pseudogenes or gene fragments [58], and 1,060 (54.7%) of them are transcriptionally active [59]. While the function of pseudogenes in plant remains poorly characterized, LncRNAs condition gene expression in plants by regulating histone modification, transcription machinery, RNA processing machinery and posttranscriptional [60]. The 13,181 lncRNA transcripts, particularly the 55 lncRNA transcripts differentially expressed between root tip and mature root, are an important resource for studying lncRNA regulation of root development.

### Gene expression and root development

Although root has a much simpler anatomical structure as compared to the shoot and flower, it grows in a very different environment, underground, implying the existence of root-specific expression patterns including a set of root-specific genes. We found that 6.8% of the protein-coding genes are specifically expressed in root, not in the aboveground portion of wheat plants. Further characterization of these root-specific genes using reverse genetics approaches will shed new light on root development.

Current assembly of wheat root transcriptome contains 91,543 HC protein-coding transcripts and 16,074 non-ORF transcripts, but only a small fraction of the transcriptome, 1.17%, was differentially expressed in the root tip and mature root tissues, similar to the result obtained in rice [61]. In rice, 1,761 of the 2,067 DETs showed higher transcription level in the mature root tissue [61]. Opposite to the finding in rice, 1,083 of 1,728 wheat DETs were up-regulated or induced in the root tips.

Root tip and mature root tissues differ in several functional aspects, and these differences are reflected at the transcriptome level. First of all, root tips contain apical meristem for maintaining cell division capacity. Consistent with this, several TFs for maintaining meristem indeterminacy, such as GRAS TFs homologous to AtHAM2 and AtHAM3 of Arabidopsis [62] and AP2 TFs homologous to AIL5 [63] and BABY BOOM [64], were up-regulated in root tips. Besides, numerous genes related auxin transport and response are up-regulated in root tips and auxin catabolic, and auxin signal suppressor genes were down-regulated in root tips. BR is critical in the regulation of cell expansion [65], and increased expression of three BR biosynthetic genes in root tips was probably due to the partial inclusion of elongation zone in the root tip samples. Another important function of root tips is to percept gravitropism, which is achieved through starch statoliths [66]. In agreement with this function, transcription of 19 starch metabolic genes was up-regulated in root tips (Fig 8). In another aspect, the matured root part mainly functions in transporting water and minerals, which is achieved by development of lateral roots, root hairs, and vascular system. For lateral root development, four lateral root-promoting TF genes including two LRP1 [67], a KUODA1 [68], and an AtNAC1 homolog, were up-regulated in the mature zone, and an AtMBY93 of Arabidopsis, a negative regulator of lateral root [69], was down-regulated in the mature zone. Increased expression of sucrose synthase in the mature zone may also be related to lateral root development as seen in soybean [70]. Another difference in the mature zone from root tips lies in the differentiation of vascular bundles. In this respect, nine lignin biosynthetic genes and a homolog of *SECONDARY WALL-ASSOCIATED NAC DOMAIN PROTEIN 2*, encoding a NAC TF activating the lignin biosynthetic genes [71], were up-regulated in the mature root portion.

Development of the root transcriptome assembly and identification of the DETs lay a foundation for molecular studies of wheat root biology and for improving soil-borne stress tolerance. In this respect, the recent development of sequence-cataloged TILLING libraries [72] will be very helpful in validating the function of DETs and homologs of root regulators identified in the model plant Arabidopsis and rice. Genome editing technologies also can be used for targeting the candidate genes in wheat for functional validation [73].

In summary, we assembled a wheat root transcriptome containing 92,335 protein-coding and 16,074 non-ORF transcripts, 6.8% and 5.2% of which, respectively, are root specific. Approximate 6.8% of coding transcripts and ~2.2% of non-ORF transcripts were not found in the current wheat genome assembly. We also identified 1,728 transcripts differentially transcribed in root tip and mature root tissues. Annotation of these DETs provides a blueprint of molecular regulation of wheat root development. Thus, they are important candidates for in-depth analysis of wheat root development by TILLING, genome editing or other reverse genetics approaches.

## Supporting information

S1 Fig Gene Ontology (GO) classification of the de novo assembled 454 contigs. (DOCX)

S2 Fig Alignment statistics of the de novo assembled root transcriptome against the genomic and the predicted cDNA sequences from the hexaploid wheat draft genome. (DOCX)

S3 Fig Gene Ontology (GO) classification of the Root transcripts with predicted ORFs. (DOCX)

S1 Table Gene Ontology (GO) classification of the contigs from de novo assembled 454 reads. (XLSX)

S2 Table Gene Ontology (GO) classification of the transcripts from illumina assembly. (XLSX)

S3 Table Transcription factor (TF) families expressed in both the root tissues used in this study. (XLSX)

S4 Table Annotation and the expression profiles of the DETs. (XLSX)

## References

[1] Petricka JJ, Winter CM, Benfey PN. Control of Arabidopsis root development. Annu Rev Plant Biol. 2012;63:563–90. 10.1146/annurev-arplant-042811-105501 ; PubMed Central PMCID: PMC3646660. 22404466 PMC3646660

[2] Hochholdinger F, Zimmermann R. Conserved and diverse mechanisms in root development. Curr Opin Plant Biol. 2008;11(1):70–4. 10.1016/j.pbi.2007.10.002 18006363

[3] Rebouillat J, Dievart A, Verdeil JL, Escoute J, Giese G, Breitler JC, et al Molecular Genetics of Rice Root Development. Rice. 2008;2(1):15–34. 10.1007/s12284-008-9016-5

[4] Coudert Y, Perin C, Courtois B, Khong NG, Gantet P. Genetic control of root development in rice, the model cereal. Trends Plant Sci. 2010;15(4):219–26. 10.1016/j.tplants.2010.01.008 . 20153971

[5] Strickler SR, Bombarely A, Mueller LA. Designing a transcriptome next-generation sequencing project for a nonmodel plant species. Am J Bot. 2012;99(2):257–66. 10.3732/ajb.1100292 22268224

[6] Brenchley R, Spannagl M, Pfeifer M, Barker GL, D'Amore R, Allen AM, et al Analysis of the bread wheat genome using whole-genome shotgun sequencing. Nature. 2012;491(7426):705–10. 10.1038/nature11650 23192148 PMC3510651

[7] The International Wheat Genome Sequencing Consortium. A chromosome-based draft sequence of the hexaploid bread wheat (*Triticum aestivum*) genome. Science. 2014;345(6194). 10.1126/science.1251788 25035500

[8] Ling HQ, Zhao S, Liu D, Wang J, Sun H, Zhang C, et al Draft genome of the wheat A-genome progenitor *Triticum urartu*. Nature. 2013;496(7443):87–90. 10.1038/nature11997 23535596

[9] Jia J, Zhao S, Kong X, Li Y, Zhao G, He W, et al Aegilops tauschii draft genome sequence reveals a gene repertoire for wheat adaptation. Nature. 2013;496(7443):91–5. 10.1038/nature12028 . 23535592

[10] Li HZ, Gao X, Li XY, Chen QJ, Dong J, Zhao WC. Evaluation of assembly strategies using RNA-seq data associated with grain development of wheat (Triticum aestivum L.). PLoS One. 2013;8(12):e83530 10.1371/journal.pone.0083530 ; PubMed Central PMCID: PMC3861526. 24349528 PMC3861526

[11] Camilios-Neto D, Bonato P, Wassem R, Tadra-Sfeir MZ, Brusamarello-Santos LC, Valdameri G, et al Dual RNA-seq transcriptional analysis of wheat roots colonized by *Azospirillum brasilense* reveals up-regulation of nutrient acquisition and cell cycle genes. BMC Genomics. 2014;15:378 10.1186/1471-2164-15-378 24886190 PMC4042000

[12] Kumar RR, Goswami S, Sharma SK, Kala YK, Rai GK, Mishra DC, et al Harnessing next generation sequencing in climate change: RNA-Seq analysis of heat stress-responsive genes in wheat (*Triticum aestivum* L.). OMICS. 2015;19(10):632–47. 10.1089/omi.2015.0097 26406536 PMC4615779

[13] Pearce S, Kippes N, Chen A, Debernardi JM, Dubcovsky J. RNA-seq studies using wheat PHYTOCHROME B and PHYTOCHROME C mutants reveal shared and specific functions in the regulation of flowering and shade-avoidance pathways. BMC Plant Biol. 2016;16(1):141 10.1186/s12870-016-0831-3 27329140 PMC4915087

[14] Leach LJ, Belfield EJ, Jiang C, Brown C, Mithani A, Harberd NP. Patterns of homoeologous gene expression shown by RNA sequencing in hexaploid bread wheat. BMC Genomics. 2014;15:276 10.1186/1471-2164-15-276 24726045 PMC4023595

[15] Seifert F, Bössow S, Kumlehn J, Gnad H, Scholten S. Analysis of wheat microspore embryogenesis induction by transcriptome and small RNA sequencing using the highly responsive cultivar "Svilena". BMC Plant Biol. 2016;16:97 10.1186/s12870-016-0782-8 27098368 PMC4839079

[16] Zhang S, Song G, Gao J, Li Y, Guo D, Fan Q, et al Transcriptome characterization and differential expression analysis of cold-responsive genes in young spikes of common wheat. J Biotechnol. 2014;189:48–57. 10.1016/j.jbiotec.2014.08.032 25240441

[17] Ho1fstad AN, Nussbaumer T, Akhunov E, Shin S, Kugler KG, Kistler HC, Mayer KF, Muehlbauer GJ. Examining the transcriptional response in wheat near-isogenic lines to infection and deoxynivalenol treatment. Plant Genome. 2016;9(1):1–15. 10.3835/plantgenome2015.05.0032 27898755

[18] Feng Y, Zhao Y, Wang K, Li YC, Wang X, Yin J. Identification of vernalization responsive genes in the winter wheat cultivar Jing841 by transcriptome sequencing. J Genet Genomics. 2016;95(4):957–64. 10.1007/s12041-016-0724-0 27994195

[19] Cantu D, Pearce SP, Distelfeld A, Christiansen MW, Uauy C, Akhunov E, et al Effect of the down-regulation of the high Grain Protein Content (GPC) genes on the wheat transcriptome during monocarpic senescence. BMC Genomics. 2011;12:492 10.1186/1471-2164-12-492 ; PubMed Central PMCID: PMC3209470. 21981858 PMC3209470

[20] Rangan P, Furtado A, Henry RJ. The transcriptome of the developing grain: a resource for understanding seed development and the molecular control of the functional and nutritional properties of wheat. BMC Genomics. 2017;18(1):766 10.1186/s12864-017-4154-z 29020946 PMC5637334

[21] Ma J, Li R, Wang H, Li D, Wang X, Zhang Y, et al Transcriptomics analyses reveal wheat responses to drought stress during reproductive stages under field conditions. Front Plant Sci. 2017;8:592 10.3389/fpls.2017.00592 28484474 PMC5399029

[22] Powell JJ, Carere J, Fitzgerald TL, Stiller J, Covarelli L, Xu Q, et al The Fusarium crown rot pathogen Fusarium pseudograminearum triggers a suite of transcriptional and metabolic changes in bread wheat (*Triticum aestivum* L.). Ann Bot. 2017;119(5):853–67. 10.1093/aob/mcw207 27941094 PMC5604588

[23] Xing P, Zhang X, Bao Y, Wang Y, Wang H, Li X. Comparative transcriptome analyses of resistant and susceptible near-isogenic wheat lines following inoculation with *Blumeria graminis* f. sp. *tritici*. Int J Genomics. 2017;2017:7305684 10.1155/2017/7305684 28553643 PMC5434243

[24] Zhang Q, Xia C, Zhang L, Dong C, Liu X, Kong X. Transcriptome analysis of a premature leaf senescence mutant of common wheat (*Triticum aestivum* L.). Int J Mol Sci. 2018;19(3): E782 10.3390/ijms19030782 29534430 PMC5877643

[25] Biselli C, Bagnaresi P, Faccioli P, Hu X, Balcerzak M, Mattera MG, et al Comparative transcriptome profiles of near-isogenic hexaploid wheat lines differing for effective alleles at the 2DL FHB resistance QTL. Front Plant Sci. 2018;9:37 10.3389/fpls.2018.00037 29434615 PMC5797473

[26] Liu YJ, Gao SQ, Tang YM, Gong J, Zhang X, Wang YB, et al Transcriptome analysis of wheat seedling and spike tissues in the hybrid Jingmai 8 uncovered genes involved in heterosis. Planta. 2018;247(6):1307–21. 10.1007/s00425-018-2848-3 29504038

[27] Chu Z, Chen J, Sun J, Dong Z, Yang X, Wang Y, et al *De novo* assembly and comparative analysis of the transcriptome of embryogenic callus formation in bread wheat (*Triticum aestivum* L.). BMC Plant Biol. 2017;17(1):224 10.1186/s12870-017-1166-4 29258440 PMC5735865

[28] Hu L, Xie Y, Fan S, Wang Z, Wang F, Zhang B, et al Comparative analysis of root transcriptome profiles between drought-tolerant and susceptible wheat genotypes in response to water stress. Plant Sci. 2018;272(276–293). 10.1016/j.plantsci.2018.03.036 29807601

[29] Patel RK, Jain M. NGS QC Toolkit: a toolkit for quality control of next generation sequencing data. PLoS One. 2012;7(2):e30619 10.1371/journal.pone.0030619 22312429 PMC3270013

[30] Lohse M, Bolger AM, Nagel A, Fernie AR, Lunn JE, Stitt M, et al RobiNA: a user-friendly, integrated software solution for RNA-Seq-based transcriptomics. Nucleic Acids Res. 2012;40(Web Server issue):W622–7. Epub 2012 Jun 8. 10.1093/nar/gks540 22684630 PMC3394330

[31] Schmieder R, Edwards R. Quality control and preprocessing of metagenomic datasets. Bioinformatics. 2011;27(6):863–4. 10.1093/bioinformatics/btr026 21278185 PMC3051327

[32] Schmieder R, Lim YW, Edwards R. Identification and removal of ribosomal RNA sequences from metatranscriptomes. Bioinformatics. 2012;28(3):433–5. 10.1093/bioinformatics/btr669 22155869 PMC3268242

[33] Pertea G, Huang X, Liang F, Antonescu V, Sultana R, Karamycheva S, et al TIGR Gene Indices clustering tools (TGICL): a software system for fast clustering of large EST datasets. Bioinformatics. 2003;19(5):651–2. 12651724 10.1093/bioinformatics/btg034

[34] Chevreux B, Pfisterer T, Drescher B, Driesel AJ, Muller WE, Wetter T, et al Using the miraEST assembler for reliable and automated mRNA transcript assembly and SNP detection in sequenced ESTs. Genome Res. 2004;14(6):1147–59. 10.1101/gr.1917404 ; PubMed Central PMCID: PMC419793. 15140833 PMC419793

[35] Huang X, Madan A. CAP3: A DNA sequence assembly program. Genome Res. 1999;9(9):868–77. 10508846 10.1101/gr.9.9.868 PMC310812

[36] Schulz MH, Zerbino DR, Vingron M, Birney E. Oases: robust de novo RNA-seq assembly across the dynamic range of expression levels. Bioinformatics. 2012;28(8):1086–92. 10.1093/bioinformatics/bts094 22368243 PMC3324515

[37] Robertson G, Schein J, Chiu R, Corbett R, Field M, Jackman SD, et al *De novo* assembly and analysis of RNA-seq data. Nat Methods. 2010;7(11):909–12. 10.1038/nmeth.1517 20935650

[38] Li W, Godzik A. Cd-hit: a fast program for clustering and comparing large sets of protein or nucleotide sequences. Bioinformatics. 2006;22(13):1658–9. 10.1093/bioinformatics/btl158 . 16731699

[39] Khan Z, Bloom JS, Kruglyak L, Singh M. A practical algorithm for finding maximal exact matches in large sequence datasets using sparse suffix arrays. Bioinformatics. 2009;25(13):1609–16. 10.1093/bioinformatics/btp275 19389736 PMC2732316

[40] Parra G, Bradnam K, Korf I. CEGMA: a pipeline to accurately annotate core genes in eukaryotic genomes. Bioinformatics. 2007;23(9):1061–7. 10.1093/bioinformatics/btm071 . 17332020

[41] Krasileva KV, Buffalo V, Bailey P, Pearce S, Ayling S, Tabbita F, et al Separating homeologs by phasing in the tetraploid wheat transcriptome. Genome Biol. 2013;14(6):R66 10.1186/gb-2013-14-6-r66 . 23800085 PMC4053977

[42] Bansal V, Bafna V. HapCUT: an efficient and accurate algorithm for the haplotype assembly problem. Bioinformatics. 2008;24(26):i153–9. 18689818 10.1093/bioinformatics/btn298

[43] Zhang C, Zhang B, Lin L-L, Zhao S. Evaluation and comparison of computational tools for RNA-seq isoform quantification. BMC Genomics. 2017;18:583 10.1186/s12864-017-4002-1 28784092 PMC5547501

[44] Lohse M, Nagel A, Herter T, May P, Schroda M, Zrenner R, et al Mercator: a fast and simple web server for genome scale functional annotation of plant sequence data. Plant Cell Environ. 2014;37(5):1250–8. 10.1111/pce.12231 24237261

[45] Usadel B, Poree F, Nagel A, Lohse M, Czedik-Eysenberg AS, M. A guide to using MapMan to visualize and compare Omics data in plants: a case study in the crop species, Maize. Plant Cell Environ. 2009;32(9):1211–29. 10.1111/j.1365-3040.2009.01978.x 19389052

[46] Benkova E, Hejatko J. Hormone interactions at the root apical meristem. Plant Mol Biol. 2009;69(4):383–96. 10.1007/s11103-008-9393-6 . 18807199

[47] Choe S, Dilkes BP, Gregory BD, Ross AS, Yuan H, Noguchi T, et al The Arabidopsis dwarf1 mutant is defective in the conversion of 24-methylenecholesterol to campesterol in brassinosteroid biosynthesis. Plant Physiol. 1999;119(3):897–907. 10069828 10.1104/pp.119.3.897 PMC32104

[48] Wasternack C. Jasmonates: An update on biosynthesis, signal transduction and action in plant stress response, growth and developmen. Ann Bot. 2007;100(4):681–97. 10.1093/aob/mcm079 17513307 PMC2749622

[49] Kataoka T, Hayashi N, Yamaya T, Takahashi H. Root-to-shoot transport of sulfate in Arabidopsis: evidence for the role of SULTR3;5 as a component of low-affinity sulfate transport system in the root vasculature. Plant Physiol. 2004;136:4198–204. 10.1104/pp.104.045625 15531709 PMC535849

[50] Rosado A, Schapire AL, Bressan RA, Harfouche AL, Hasegawa PM, Valpuesta V, et al The Arabidopsis tetratricopeptide repeat-containing protein TTL1 is required for osmotic stress responses and abscisic acid sensitivity. Plant Physiol. 2006;142(3):1113–26. 10.1104/pp.106.085191 16998088 PMC1630727

[51] Zilberman D, Cao X, Jacobsen SE. ARGONAUTE4 control of locus-specific siRNA accumulation and DNA and histone methylation. Science. 2003;299(5607):716–9. 10.1126/science.1079695 12522258

[52] Oono Y, Kobayashi F, Kawahara Y, Yazawa T, Handa H, Itoh T, et al Characterisation of the wheat (*Triticum aestivum* L.) transcriptome by de novo assembly for the discovery of phosphate starvation-responsive genes: gene expression in Pi-stressed wheat. BMC Genomics. 2013;14:77 10.1186/1471-2164-14-77 ; PubMed Central PMCID: PMC3598684. 23379779 PMC3598684

[53] Yang L, Xie L, Xue B, Goodwin PH, Quan X, Zheng C, et al Comparative transcriptome profiling of the early infection of wheat roots by *Gaeumannomyces graminis* var. *tritici*. PLoS One. 2015;10(4):e0120691 10.1371/journal.pone.0120691 25875107 PMC4397062

[54] Bedada G, Westerbergh A, Müller T, Galkin E, Bdolach E, Moshelion M, et al Transcriptome sequencing of two wild barley (*Hordeum spontaneum* L.) ecotypes differentially adapted to drought stress reveals ecotype-specific transcripts. BMC Genomics. 2014;15:995 10.1186/1471-2164-15-995 25408241 PMC4251939

[55] Reeksting BJC, N., Mahomed W, Engelbrecht J, van den Berg N. De novo sequencing, assembly, and analysis of the root transcriptome of Persea americana (Mill.) in response to Phytophthora cinnamomi and flooding. PLoS One. 2014;9(2):e86399 10.1371/journal.pone.0086399 24563685 PMC3919710

[56] Bedre R, Mangu VR, Srivastava S, Sanchez LE, Baisakh N. Transcriptome analysis of smooth cordgrass (*Spartina alterniflor*a Loisel), a monocot halophyte, reveals candidate genes involved in its adaptation to salinity. BMC Genomics. 2016;17(1):657 10.1186/s12864-016-3017-3 27542721 PMC4992267

[57] Akhunov ED, Sehgal S, Liang H, Wang S, Akhunova AR, Kaur G, et al Comparative analysis of syntenic genes in grass genomes reveals accelerated rates of gene structure and coding sequence evolution in polyploid wheat. Plant Physiol. 2013;161(1):252–65. 10.1104/pp.112.205161 ; PubMed Central PMCID: PMC3532256. 23124323 PMC3532256

[58] Choulet F, Alberti A, Theil S, Glover N, Barbe V, Daron J, et al Structural and functional partitioning of bread wheat chromosome 3B. Science. 2014;345(6194):1249721 10.1126/science.1249721 25035497

[59] Pingault L, Choulet F, Alberti A, Glover N, Wincker P, Feuillet CP, E. Deep transcriptome sequencing provides new insights into the structural and functional organization of the wheat genome. Genome Biol. 2015;16:29 10.1186/s13059-015-0601-9 25853487 PMC4355351

[60] Liu J, Wang H, Chua NH. Long noncoding RNA transcriptome of plants. Plant Biotechnol J. 2015;13(3):319–28. 10.1111/pbi.12336 25615265

[61] Kyndt T, Denil S, Haegeman A, Trooskens G, De Meyer T, Van Criekinge W, et al Transcriptome analysis of rice mature root tissue and root tips in early development by massive parallel sequencing. J Exp Bot. 2012;63(5):2141–57. 10.1093/jxb/err435 22213813

[62] Engstrom EM, Andersen CM, Gumulak-Smith J, Hu JO, E., Sozzani R, Bowman JL. Arabidopsis homologs of the petunia hairy meristem gene are required for maintenance of shoot and root indeterminacy. Plant Physiol. 2011;155(2):735–50. 10.1104/pp.110.168757 21173022 PMC3032463

[63] Nole-Wilson S, Tranby TL, Krizek BA. AINTEGUMENTA-like (AIL) genes are expressed in young tissues and may specify meristematic or division-competent states. Plant Mol Biol. 2005;57(5):613–28. 10.1007/s11103-005-0955-6 15988559

[64] Galinha C, Hofhuis H, Luijten M, Willemsen V, Blilou I, Heidstra R, et al PLETHORA proteins as dose-dependent master regulators of Arabidopsis root development. Nature. 2007;449(7165):1053–7. 10.1038/nature06206 . 17960244

[65] Jaillais Y, Vert G. Brassinosteroid signaling and BRI1 dynamics went underground. Curr Opin Plant Biol. 2016;33:92–100. 10.1016/j.pbi.2016.06.014 27419885 PMC5055102

[66] Fitzelle K, J., Kiss JZ. Restoration of gravitropic sensitivity in starch-deficient mutants of Arabidopsis by hypergravity. J Exp Bot. 2001;52(355):265–75. 11283171

[67] Smith DL, Fedoroff NV. LRP1, a gene expressed in lateral and adventitious root primordia of arabidopsis. Plant Cell. 1995;7(6):735–45. 10.1105/tpc.7.6.735 7647564 PMC160824

[68] Lu D, T W, Persson S, Mueller-Roeber B, Schippers JH. Transcriptional control of ROS homeostasis by KUODA1 regulates cell expansion during leaf development. Nat Commun. 2014;5:3767 10.1038/ncomms4767 PMC4024751 24806884

[69] Gibbs DJ, Coates JC. AtMYB93 is an endodermis-specific transcriptional regulator of lateral root development in arabidopsis. Plant Signal Behav. 2014;9(10):e970406 10.4161/15592316.2014.970406 25482809 PMC4622915

[70] Liu W, Han X, Zhan G, Zhao Z, Feng Y, Wu C. A novel sucrose-regulatory mads-box transcription factor gmnmhc5 promotes root development and nodulation in soybean (*Glycine max* [L.] Merr.). Int J Mol Sci. 2015;16(9):20657–73. 10.3390/ijms160920657 26404246 PMC4613224

[71] Hussey SG, Mizrachi E, Spokevicius AV, Bossinger G, Berger DK, Myburg AA. SND2, a NAC transcription factor gene, regulates genes involved in secondary cell wall development in Arabidopsis fibres and increases fibre cell area in Eucalyptus. BMC Plant Biol. 2011;11:173 10.1186/1471-2229-11-173 22133261 PMC3289092

[72] Krasileva KV, Vasquez-Gross HA, Howell T, Bailey P, Paraiso F, Clissold L, et al Uncovering hidden variation in polyploid wheat. Proc Natl Acad Sci U S A. 2017;114(6):E913–E21. 10.1073/pnas.1619268114 28096351 PMC5307431

[73] Wang Y, Cheng X, Shan Q, Zhang Y, Liu J, Gao C, et al Simultaneous editing of three homoeoalleles in hexaploid bread wheat confers heritable resistance to powdery mildew. Nature Biotechnology. 2014;32(9):947–51. 10.1038/nbt.2969 25038773

