# Supplementary material for: De novo assembly of wheat root transcriptomes and transcriptional signature of longitudinal differentiation
Source: PLoS One. 2018 Nov 5;13(11):e0205582. doi: 10.1371/journal.pone.0205582 (PMC6218025; doi:10.1371/journal.pone.0205582)

**S1 Fig.** Gene Ontology (GO) classification of the de novo assembled 454 contigs. (A) Biological processes (B) Molecular functions (C) Subcellular localization. The GO categories are indicated on the X-axis, and the number of transcripts in each category is indicated on the Y-axis.


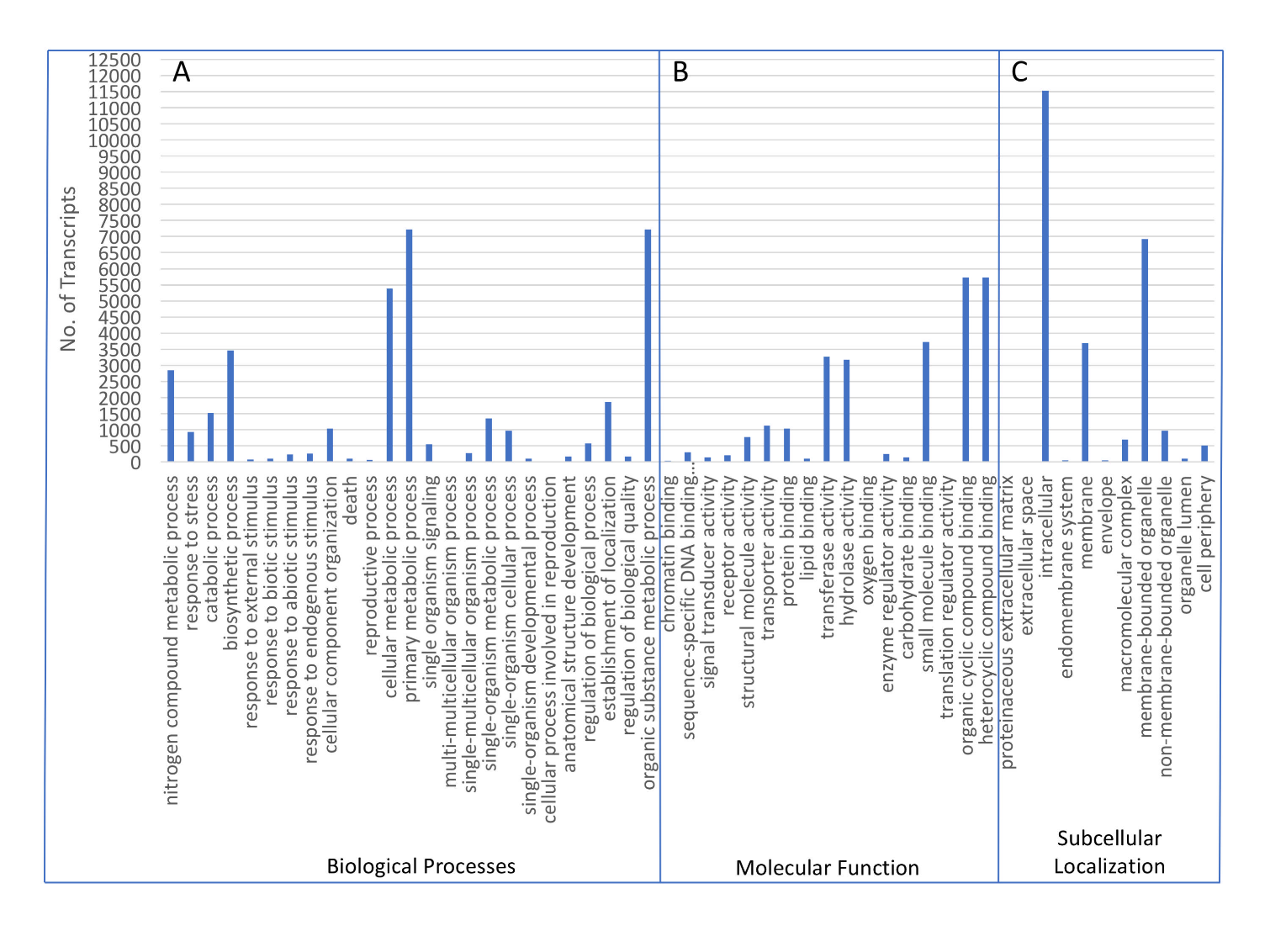

Supplement: S1 Fig — (DOCX) [file pone.0205582.s001.docx]
