# Supplementary material for: De novo assembly of wheat root transcriptomes and transcriptional signature of longitudinal differentiation
Source: PLoS One. 2018 Nov 5;13(11):e0205582. doi: 10.1371/journal.pone.0205582 (PMC6218025; doi:10.1371/journal.pone.0205582)

**S2 Fig.** Alignment statistics of the de novo assembled root transcriptome against the genomic and the predicted cDNA sequences from the hexaploid wheat draft genome. (A) Aclustered and stacked bar chart showing the coverage percent and the identity percent of the root transcripts with predicted ORFs against the genomic and the DNA sequences of the draft genome. (B) A clustered and a stacked bar chart showing the coverage percent and the identity percent of the root transcripts with without predicted ORFs against the genomic and the DNA sequences of the draft genome. The bars are stacked by the percent identity (represented by color) of the alignment and are clustered by the percent of the query covered in the alignment (shown by the labels on the top of each cluster). The database against which the transcripts were compared is indicated on the X-axis. The number of transcripts in each bin is indicated on the Y-axis.


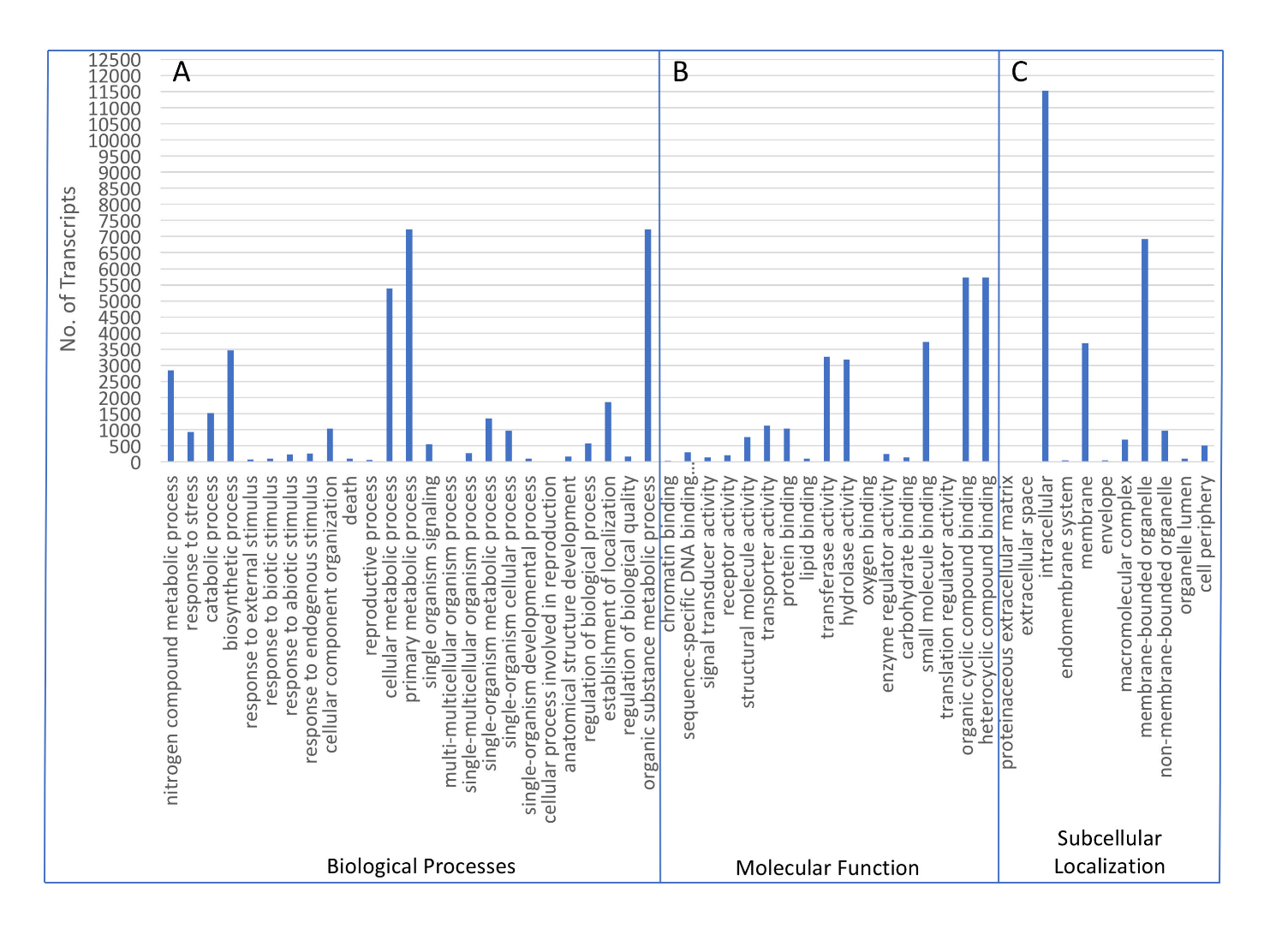

Supplement: S2 Fig — (DOCX) [file pone.0205582.s002.docx]
