# Supplementary material for: De novo assembly of wheat root transcriptomes and transcriptional signature of longitudinal differentiation
Source: PLoS One. 2018 Nov 5;13(11):e0205582. doi: 10.1371/journal.pone.0205582 (PMC6218025; doi:10.1371/journal.pone.0205582)

**S3 Fig.** Gene Ontology (GO) classification of the Root transcripts with predicted ORFs. (A) Biological processes (B) Molecular functions (C) Subcellular localization. The GO categories are indicated on the X-axis, and the number of transcripts in each category is indicated on the Y-axis.


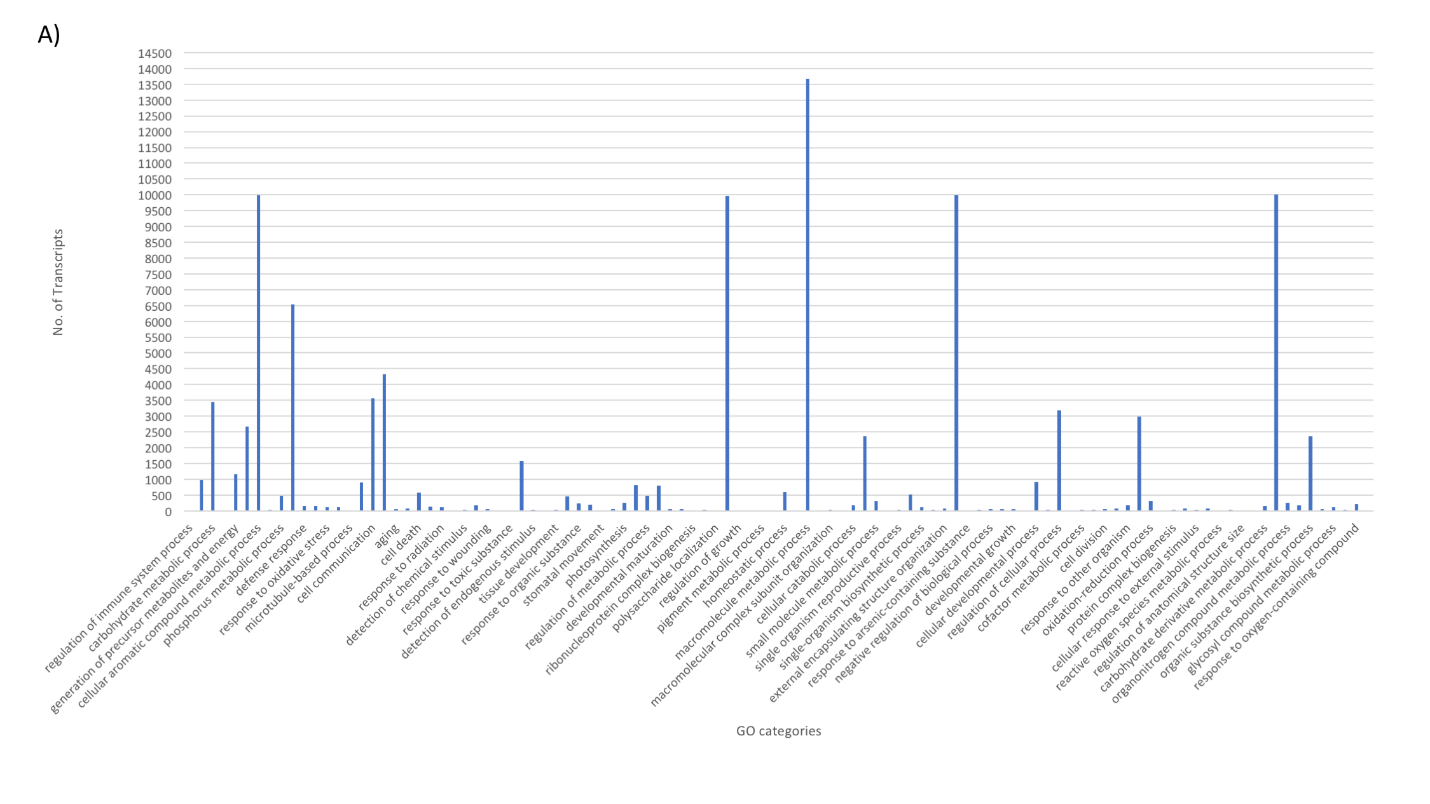


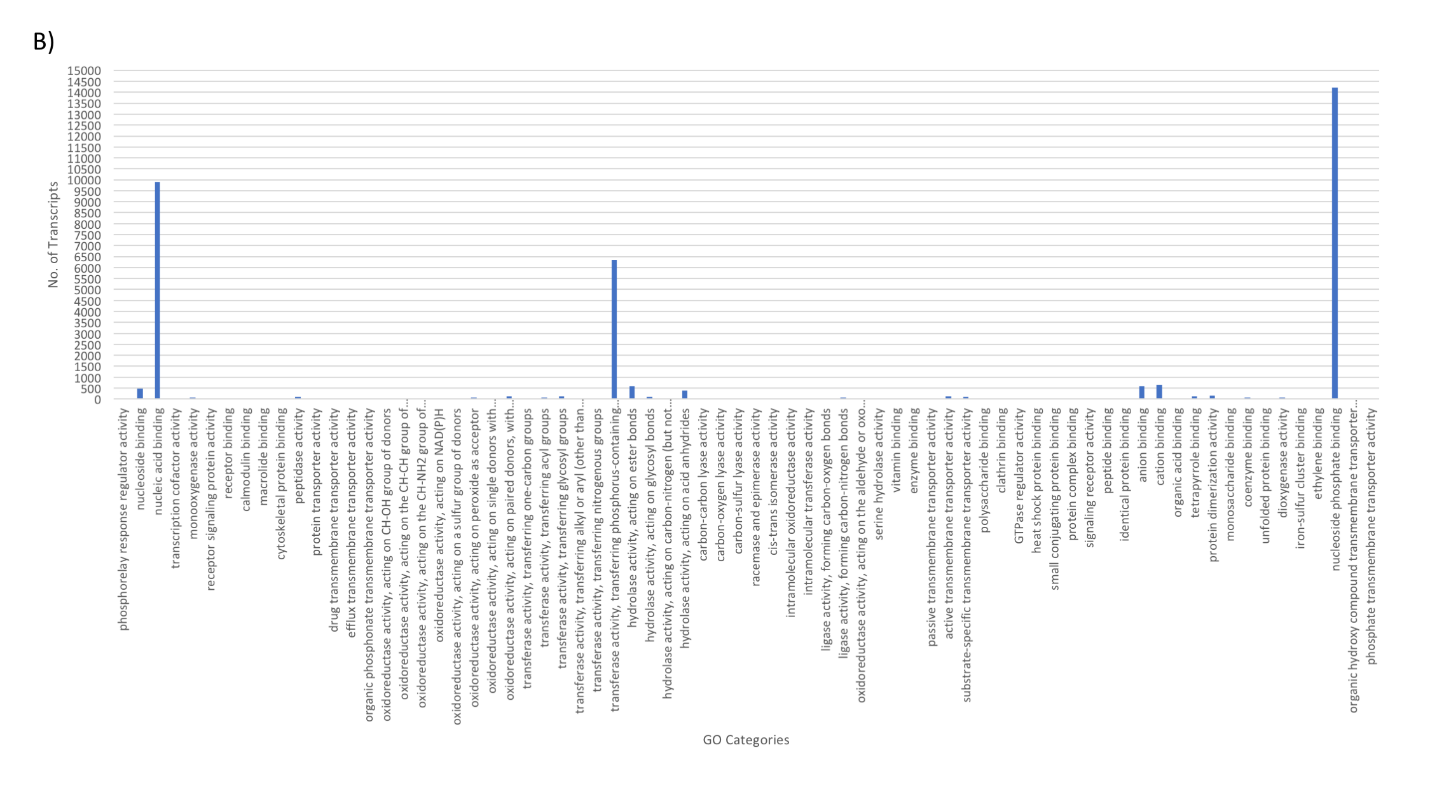


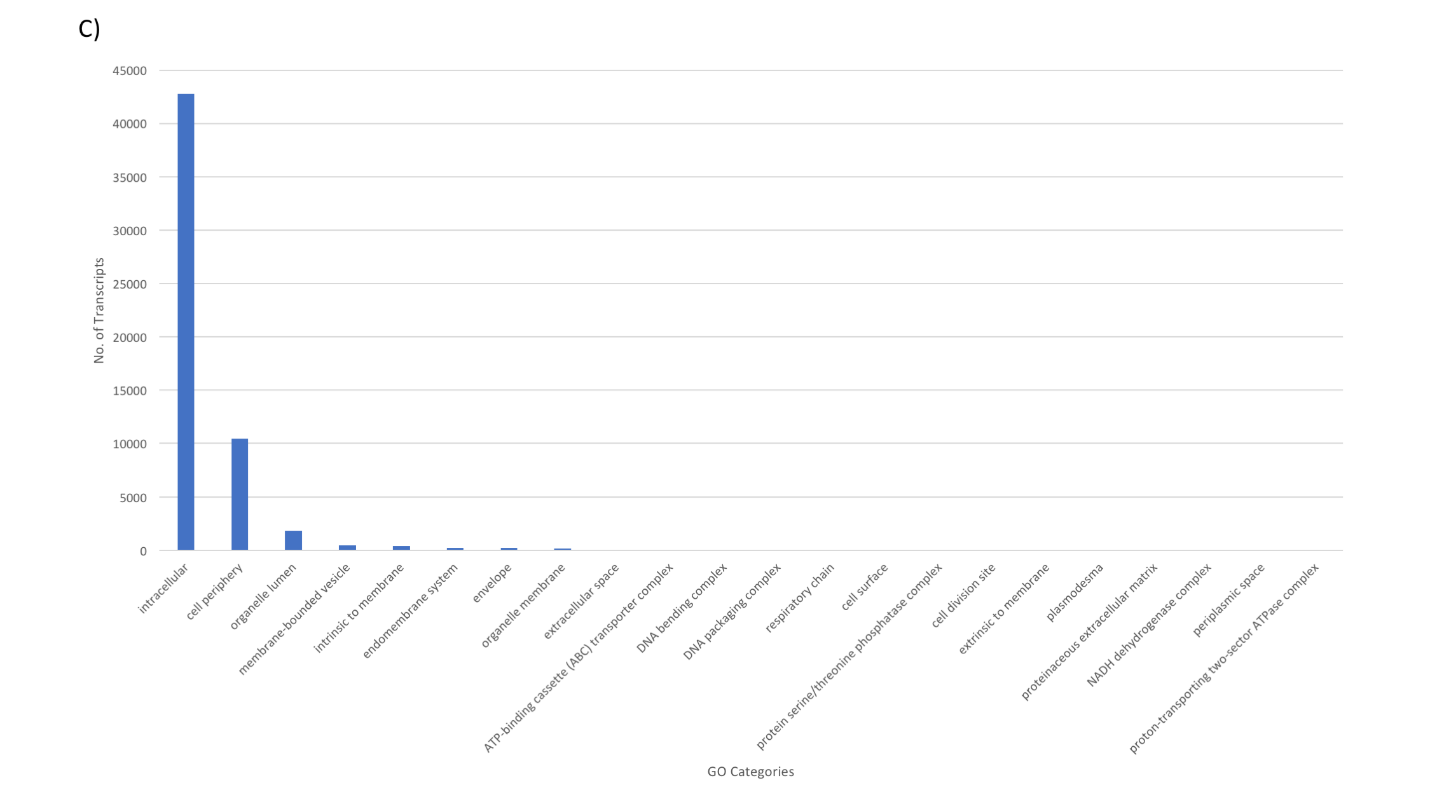

Supplement: S3 Fig — (DOCX) [file pone.0205582.s003.docx]
